# Thermal illusions for thermal displays: a review

**DOI:** 10.3389/fnhum.2023.1278894

**Published:** 2023-12-05

**Authors:** Subhankar Karmakar, Anindita Kesh, Manivannan Muniyandi

**Affiliations:** Department of Applied Mechanics and Biomedical Engineering, Indian Institute of Technology Madras, Chennai, India

**Keywords:** thermal illusions, thermal displays, psychophysics, virtual reality, haptics

## Abstract

Thermal illusions, a subset of haptic illusions, have historically faced technical challenges and limited exploration. They have been underutilized in prior studies related to thermal displays. This review paper primarily aims to comprehensively categorize thermal illusions, offering insights for diverse applications in thermal display design. Recent advancements in the field have spurred a fresh perspective on thermal and pain perception, specifically through the lens of thermal illusions.

## 1 Introduction

Thermal displays are devices that can present a desirable temperature cue to the skin. Thermal displays have the potential to serve as a significant component of virtual reality (VR) experiences. In conjunction with the visual stimulation provided by virtual reality (VR), designers are attempting to incorporate haptic and thermal feedback mechanisms with the aim of augmenting the user's level of immersion (Dionisio, 1997; Kim et al., 2020; Lee et al., 2020). Thermal displays can also be implemented to enhance the user experience in telemarketing. Gaining a more accurate perception of a product prior to making an online purchase might result in increased customer satisfaction and a decrease in the frequency of product returns. Additional uses of thermal displays encompass teleoperation, communication, and interactive art.

Several studies have been trying to model thermal displays that can transfer heat using different methods (conduction, convection, and radiation) (Zerkus et al., 1993; Caldwell et al., 1995; Oron-Gilad et al., 2008; Gallo and Bleuler, 2015; Saga, 2015; Goetz et al., 2020). These displays possess a wide range of potential uses in the modern world, which is increasingly characterized by virtualization. Thermal displays can be made more effective and efficient by implementing several thermal illusions.

The definition of “illusion” can have varying interpretations (Zavagno et al., 2015; Todorović, 2020). An extreme view is that all perceptions are illusions of some form. However, in this paper, we are considering a more accepted and practical view that defines an illusion as a mismatch between an applied stimulus and the resultant perception. Through the examination of illusions, researchers try to understand the cognitive processes that enable individuals to perceive and internally conceptualize the world around them (Lederman and Jones, 2011). Previous studies have employed illusions as a means to augment perception by compensating for the absence of certain elements in the perceptual experience. For instance, incorporating the element of stiffness in a visually simulated virtual spring can elicit haptic perceptions of physical resistance (Lécuyer, 2009). Thermal illusions are a subset of haptic illusions that involve the perception of temperature. Thermal illusions refer to phenomena in which either an individual's thermal perception becomes distorted or the application of a thermal stimulus leads to the distortion of another perceptual modality. Thermal illusions can aid in the development of multisensory displays that include multiple sensory modalities to enhance the overall experience.

Different modalities can help in conveying temperature awareness in humans. Visual, auditory, and tactile stimuli can send separate but complementary information, contributing to the development of a cohesive perception (Ho et al., 2014). As an illustration, during the process of boiling water, the auditory perception of the high-pitched sound emitted by the teapot when it attains the boiling point of 100°C, together with the visual observation of the emission of steam from the spout, serve as perceptual cues that provide information regarding the temperature. Nevertheless, the temperature is really perceived when one physically holds the handle of the teapot. When an object is touched, information about its texture, warmth, and other properties is encoded, resulting in the object's perceptual experience (Ino et al., 1993). The perception of touch is generated through the stimulation of specific cutaneous mechanoreceptors, namely, Merkel disks, Ruffini cylinders, Meissner corpuscles, Pacinian corpuscles, and hair follicle receptors. Additionally, Paciniform corpuscles, Meissnerform corpuscles, and free nerve endings, including C-touch, C-HTMR, and Aδ-HTMR, contribute to this sensory experience. They all respond to unique mechanical stimuli (DeMyer, 1988).

This paper aims to review different types of thermal illusions and discuss how they can be used to improve thermal displays. The implementation of improved thermal displays has the potential to enhance the user experience in virtual reality (VR) environments and touch-based communication devices. Enhanced thermal displays can potentially provide benefits to the sphere of telemarketing. The utilization of thermal displays and greater comprehension of thermal illusions can also facilitate advancements in medical research. A detailed overview of the existing thermal illusions is missing in the literature. In this review, we first provide a historical perspective and a physiological understanding of thermal perception. Later, we describe the psychophysical studies on thermal illusions and the proposed mechanisms behind them. We present two types of classification of the thermal illusions to aid in developing a categorical understanding of the illusory thermal phenomena. Furthermore, the literature also lacks a review of the different thermal displays developed by researchers along with the thermal transmission methods used by them to induce the desired thermal perception. In the current review, we look at some of the techniques used by thermal displays to create a thermal sensation and classify them based on the method of heat transfer implemented. Finally, we explored the feasibility that thermal illusions could contribute to improving the performance of thermal displays and have a range of applications.

## 2 History of research on thermal perception

A historical perspective on thermal perception research can help us understand the motivation behind thermosensation studies (Table 1). This section will provide a brief overview of the gradual development of research related to the temperature sense.

**Table 1:** Chronology of historical events in thermal perception research.

**Year**	**Discovery or invention in the field of thermal perception**
1796 A.D.	Erasmus Darwin recognized a separate sense named “heat sense” apart from the five generally recognized senses (vision, hearing, taste, smell, and touch) (Darwin, 1809)
1834	Weber observed that we perceive an increase or decrease in temperature and not the absolute temperature (Weber, 1834)
1837	Hardy and Oppel demonstrated “spatial summation” for temperature perception (Hardy et al., 1938)
1846	Weber argued that the several “qualities” of touch are interdependent and demonstrated the “Thaler illusion” (later called temperature-weight illusion) to support his claim (Weber, 1846)
1879	Ewald Hering introduced the concept of “physiological zero” which is the temperature at which the skin perceives neutral (neither hot nor cold) (Stevens and Green, 1996)
1882–1885	Sensory spots discovered independently by Blix, Goldscheider, and Donaldson (Blix, 1884; Goldscheider, 1884; Donaldson, 1885)
1895	von Frey demonstrated “paradoxical cold” or the elicitation of cold sensation at temperatures slightly below the thermal pain threshold (Boring, 1942)
1896	Thunberg demonstrated the thermal grill illusion (synthetic heat) (Craig and Bushnell, 1994)
1898	Alrutz proposed that the fusion of warm and cold causes pain in synthetic heat (Alrutz, 1898)
After 1930	Electromagnetic radiation started being used as a thermal stimulus. It helped to induce thermal sensation without concomitant mechanical stimulation (Stevens, 1983)
1935	Zotterman first identified a specific thermoreceptor (cold receptor) by implementing electrophysiological methods (Zotterman, 1935)
1937–1941	J.D. Hardy and his associates implemented radiation to measure absolute and differential thresholds of temperature as a function of different variables (Hardy et al., 1938)
1942	Boring differentiated the tactile sensation (includes temperature sense) into four categories (physiological, functional, qualitative, and perceptual) (Boring, 1942)
1951–1969	Hensel and his associates discovered certain receptors in cats, monkeys, and humans that respond to both mechanical and thermal stimuli (Hensel and Boman, 1960)
1952	Zotterman studied the spike frequency of a single warm fiber and a single cold fiber (from cats' tongue) as a function of temperature (Zotterman, 1953)
1973	Hensel demonstrated the general properties (static and dynamic response to temperature) of cold and warm receptors (Hensel, 1973)
1997	Caterina, Julius, and colleagues discovered the first heat-sensitive transient receptor potential (TRP), the capsaicin receptor (TRPV1) (Caterina et al., 1997)
2005–2008	Patapoutian, Julius, Caterina, Nilius, and others discovered more “thermoTRPs” (Patapoutian, 2005; Bandell et al., 2007; Caterina, 2007; Talavera et al., 2008)

### 2.1 Recognition of the thermal sense

Before the mid-nineteenth century, the skin was rarely thought of as an organ with several sensory modalities. The five generally recognized senses were vision, smell, hearing, taste, and touch (Stevens and Green, 1996). In 1796, Erasmus Darwin postulated the presence of more senses in his book “Zoonomia,” in addition to these five senses. Such a sense was the sensation of temperature, which he called a “heat sense” (Darwin, 1809).

In his publication named “Der Tastsinn und das Gemeingefuhl” (“Touch and Common Sensibility”), Weber (1846) first differentiated “touch” into several qualities. He differentiated between the skin's capacity to detect changes in pressure and temperature and its ability to localize sensations on the body. Weber argued that these qualities are interdependent, and to back up his assertions, he employed the “Thaler illusion,” in which a cold silver coin on the forehead seems heavier than a similar coin at a neutral temperature (neither warm nor cold) (Weber, 1846; Stevens and Green, 1996).

According to the German physiologist Ewald Hering, warmth and cold are intertwined with each other in the form of a “single thermal sense” (Boring, 1942). He claimed that warmth and cold are “opponent processes” and the same temperature might feel warm or cold depending on the Nullpunkt, or the “physiological zero,” the temperature that feels neutral (Stevens and Green, 1996).

### 2.2 Discovery of sensory spots

Sensory spots are a key discovery in the domain of thermal perception (Stevens and Green, 1996). A sensory spot is a small area of skin that produces a sensation when stimulated by pain, pressure, or temperature. Between 1882 and 1885, three independent laboratories, one led by Magnus Blix in Sweden, one by Alfred Goldscheider in Germany, and one by Henry Donaldson in America, were engaged in the discovery of these sensory spots (Blix, 1884; Goldscheider, 1884; Donaldson, 1885). Blix observed that electrical stimulation of different sites of the skin can make people feel warm or cold. He mapped the warm and cold sensitive spots using a temperature stimulator made out of a hollow metal cone. Blix's findings were validated and expanded upon by Donaldson. Goldsheider also mapped cold, warm, and pressure sensory spots, concluding that they were all distinct characteristics within the tactile modality. The mappings of the sensory spots revealed that the cold spots outnumber the warm spots by a substantial margin. In addition, the concentration of cold and warm spots varied significantly across the body. The number of cold spots on the lips is significantly higher than on the sole, while the number of warm spots on the fingers is much higher than on the dorsal side of the upper arm (Strughold, 1931).

Spatial summation refers to the phenomenon wherein the perceived magnitude of a sensation increases as the area of skin stimulation becomes broader. Hardy et al. (1938) conducted studies aimed at comprehending the phenomenon of spatial summation in relation to temperature stimuli. Spatial summation will be discussed in more detail later in this paper. The lack of warm spots in some parts of the body does not necessarily indicate insensitivity to warm stimuli. In sites characterized by a small number of warm spots, the application of a stimulus with a greater area of contact (ranging from 1 to 2 cm^2^) compared to the conventional spot mapping technique (often utilizing a surface area of 1–2 mm^2^) might induce the sensation of warmth. Therefore, it can be asserted that thermoreceptors and temperature spots are not synonymous terms. It is possible that the number of receptors exceeds the number of spots. A sensation might be produced by the simultaneous activation of several of these receptors (Stevens and Green, 1996). In addition, Maximilian von Frey made the inclusion of pain spots and paradoxical cold spots to the list of sensory spots. Paradoxical cold spots generate cold sensations even at warm temperatures between 45 and 50°C (Boring, 1942). Further technological improvements paved the way for electrophysiological research on thermoreceptors, providing a clearer idea.

### 2.3 Electrophysiological studies of thermoreceptors

Thermoreceptors are sensory units responsible for conveying temperature information from the skin to the brain. They are present in the dermal and epidermal skin layers (Hensel, 1974). The electrophysiological recordings of the individual thermoreceptor fibers helped gain insights regarding the neurophysiology behind temperature sensing (Stevens and Green, 1996). Radiometry advancements enabled the use of electromagnetic radiation as a stimulus for warmth or pain and helped to eliminate the mechanical stimulation that had been present in prior studies (Stevens, 1983). This led to a more effective stimulus for evaluating warmth and pain. Recordings of neural impulses in the cutaneous nerve fibers were done for different species, including human beings.

Different kinds of nerve fibers were found to respond differently. Some nerve fibers responded to temperature alone, whereas others to both temperature and mechanical stimuli. One type of fiber showed an overshoot of spike rate when the skin was heated and a transient inhibition of cooling the skin. On the other hand, certain fibers showed an increase in the rate of impulses when they were cooled and a brief inhibition when they were warmed (Hensel, 1973; Hallin et al., 1982). Further research has aided our understanding of the physiology of the nerve fibers involved in temperature sensing as well as the broader mechanisms at work.

## 3 Physiology and mechanisms of thermal perception

The physiology of temperature perception can be understood from different perspectives. Electrophysiological studies were conducted to observe the thermoreceptors and the afferent fibers involved in thermoreception. This section will go through these aspects in order to better comprehend the thermal illusions that will be discussed in the following sections.

### 3.1 Thermoreceptors

The discovery of thermoreceptors and their physiology has helped researchers gain insights into the fundamental processes that govern temperature perception. Thermoreceptors are sensory units that are located beneath the skin throughout the body and are stimulated by temperature changes. In fact, there are different types of receptors for the conduction of warm and cold sensations, namely, “warm” and “cold” receptors, respectively (Stevens, 2013). Thermal information is transported from thermoreceptors to the cortex in the somatosensory area 1 (S1) through the spinothalamic pathway (Milenkovic et al., 2014). Moreover, the warm and cold receptors respond differently to different thermal stimuli (Table 2).

**Table 2:** Similarities and differences between warm and cold receptors.

	**Warm receptors**	**Cold receptors**
Static response	Shows neuronal discharge at constant temperatures	Also shows neuronal discharge at constant temperatures
Dynamic response	Firing overshoots when temperature increases and ceases when temperature decreases	Firing overshoots when temperature decreases and ceases when temperature increases
Chemical stimulation	Can be chemically stimulated using camphor, capsaicin, etc.	Can be chemically stimulated using menthol, icilin, etc.
Paradoxical firing	Does not show any paradoxical firing	Shows paradoxical firing above 45°C
Afferent fibers	C-fibers	A-MSA, HCRs, CTs, SA fibers, Aδ nociceptors, C nociceptors
Receptor density	Less dense than cold receptors. Density varies across the body	More dense than warm receptors. Density varies across the body
Conduction velocity of associated afferents	Associated C-fibers: 1–2 m/s	Associated Aδ fibers: 10–20 m/s

A-MSA are mechanosensitive Aδ afferents. HCR stands for “high threshold” cold receptor. CTs or C-tactile fibers are “low threshold” mechanoreceptors that respond to both cooling and touch and SA stands for slow adapting mechanoreceptors.

**Figure 1:**
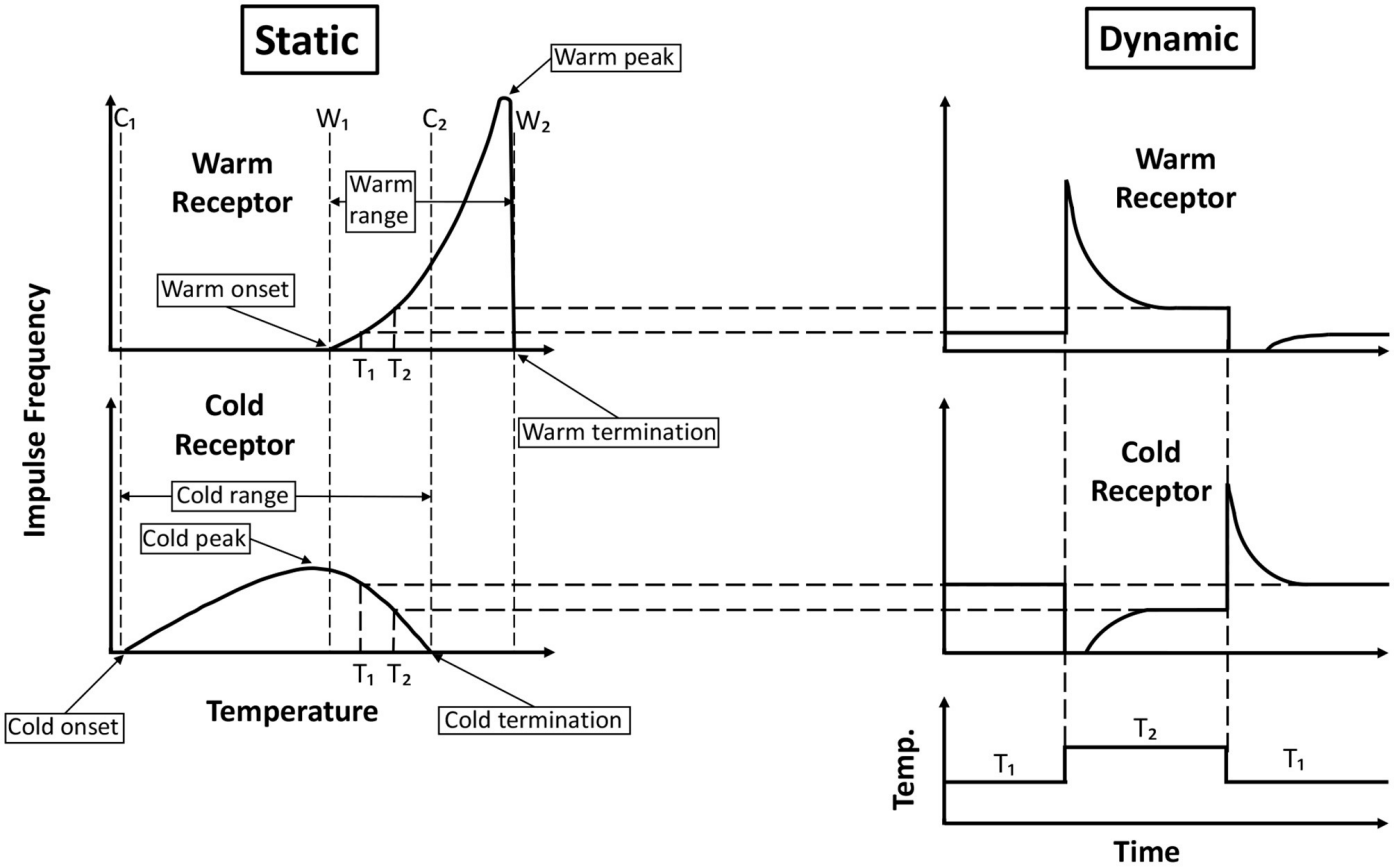
Generalized response of cold and warm receptors recorded from cat's nose. The static response is the response at a constant temperature, and the dynamic response is the response when there is a rapid change in temperature. Onset and termination of cold receptor static response are intersected by vertical lines *C*_1_ and *C*_2_, respectively. Similarly, lines *W*_1_ and *W*_2_ intersect the onset and termination of the static response of warm receptors. The peak of the cold static response lies very close to the line *W*_1_. There is an overlap in the static response range of the warm and cold receptors between *W*_1_ and *C*_2_. Adapted and redrawn from Hensel (1973).

Both the warm and cold receptors are spontaneously firing at normal skin temperatures, and there is no noticeable sensation due to this spontaneous activity. However, when the skin is heated, the warm receptors fire vigorously, and the cold receptors cease the spontaneous firing. Conversely, on cooling, the cold receptors show increased firing while the warm receptors stop their spontaneous firing. Hensel (1973) showed the response of cutaneous single warm and cold receptors present in the cat's nose to constant temperatures (static response) as well as to rapid changes in temperature (dynamic response) (Figure 1). Zotterman (1953) observed that the cold receptors also show a “paradoxical” firing at temperatures above 45°C. As a result, thermoreceptor activity can be conceived of as a combination of the response to a temperature value and the magnitude of change in that value.

Cold is perceived much faster than warmth due to the difference in the velocities of the afferent fibers (nerve fibers that carry information from the sensory receptors to the brain) associated with them. The conduction velocity of afferent fibers originating from warm receptors typically falls within the range of 1–2 m/s, but the conduction velocity of afferent fibers originating from cold receptors can be 10–20 m/s (Darian-Smith, 1984). Apart from these warm and cold receptors, there are pain-sensitive nociceptors that activate when a noxious thermal (painfully hot or cold) or mechanical stimulus is applied (Stevens, 2013).

Thermoreceptors can also be stimulated chemically, mimicking the perception of warmth or cold. A number of chemical substances can behave as agonists for temperature perception. For instance, menthol is known to elicit a cold sensation on the skin (Hensel, 1973). Calcium, on the other hand, can produce warmth when applied intracutaneously or intravenously. Calcium and chloroform both have the ability to lower warm thresholds (Schreiner, 1936; Schmidt, 1949). Carbon dioxide and carbonic acid, when applied locally, also produce warmth (Liljestrand and Magnus, 1922; Gollwitzer-Meier, 1937). Certain spices, such as capsaicine, undecylenic acid vanillylamide, and cinnamonylic-acrylic acid piperidide can induce hot as well as pain sensations (Stary, 1925; Sans, 1949). The neuronal activities in the thermoreceptors are mediated by the afferent nerve fibers to the insula cortex (Craig et al., 2000).

### 3.2 Afferent nerve fibers associated with thermosensation

The majority of the afferent nerve fibers involved in temperature sensation are C fibers and A δ fibers. C fibers are unmyelinated fibers with a lower conduction velocity, whereas the Aδ fibers are thinly myelinated with a higher conduction velocity. Studies on humans have shown that C fibers are activated by cold temperature stimuli in the innocuous range (Campero et al., 2001). Apart from these C and Aδ fibers, there are “high threshold” cold receptors (HCRs) found in monkeys' skin (LaMotte and Thalhammer, 1982). “Low threshold” mechanoreceptors termed C tactile fibers (CTs) respond to rapid cooling as well as light touch (Kumazawa and Perl, 1977). In fact, about half of the slowly adapting mechanoreceptors (SA fibers) like Merkel discs and Ruffini endings respond to cooling stimulus from normal skin temperature to 14.5°C (Hensel and Zotterman, 1951; Duclaux and Kenshalo, 1972; Cahusac and Noyce, 2007). Warm fibers, on the other hand, are C fibers that are insensitive to mechanical stimuli (Darian-Smith et al., 1979). Apart from innocuous warmth and cold, afferent fibers are involved in painful thermal perception.

Both “cold pain” and “heat pain” are mediated by C and Aδ nociceptors (Kumazawa and Perl, 1977; LaMotte and Thalhammer, 1982; Campero et al., 1996; Simone and Kajander, 1997; Schepers and Ringkamp, 2010). “Cold pain” is felt in glabrous (non-hairy) skin at temperatures between 10 and 15°C and in hairy skin at temperatures below 18°C (Davis, 1998; Harrison and Davis, 1999).

An intriguing observation regarding “heat pain” is that it is felt twice and is called a “dual pain sensation” (Campbell and LaMotte, 1983). When noxious heat stimuli were applied to the hairy skin (of the lower arm, dorsum of the hand, and foot), they produced two distinct pain sensations (Campbell and LaMotte, 1983). The first pain was a sharp, pricking sensation that occurred almost 0.4 s after the stimulus. Again, after 1–2 s of the first pain, the second pain was felt as a dull, burning sensation. This dual pain sensation was felt at the distal extremities and tended to merge in the proximal regions of the body. Also, for glabrous skin, like the skin of the palm, the dual pain was not induced (Campbell and Meyer, 1983). This dual pain sensation indicates that two types of fibers are involved in the mediation of “heat pain.” The first pain is mediated by Aδ fibers which have faster conduction velocities, and the second pain is mediated by slower-conducting C fibers (Price et al., 1977; Campbell and LaMotte, 1983).

## 4 Psychophysics of temperature perception

Psychophysics is the study of identifying a quantitative link between an external physical stimulus and the conscious perception of the sensation induced by the stimulus (Engen, 1988). Various psychophysical studies were conducted to investigate the underlying mechanisms and unique features of thermosensation. Some properties of thermal sensation, such as the two-point discrimination threshold, thermal detection threshold, thermal adaptation, thermal spatial summation, and thermal localization, are discussed below (Stevens, 2013).

### 4.1 Two-point discrimination threshold

The two-point discrimination threshold (TPDT) is often used to measure the spatial acuity of the sensory system. TPDT is the minimum distance between two cutaneous point stimuli at which they are perceived as two separate points instead of a single point. In a recent study by Frahm and Gervasio (2021), TPDT for mechanical and thermal stimuli on the right volar forearm was compared. They observed that the TPDT varied depending on the modality (mechanical or thermal) and the intensity (innocuous or noxious) of the stimuli. The TPDT of a noxious thermal stimulus (66.9 mm) was less compared to that of an innocuous thermal stimulus (80.5 mm). On the contrary, the TPDT of a noxious mechanical stimulus (47.1 mm) was found to be greater than the innocuous one (34.7 mm) (Frahm and Gervasio, 2021).

### 4.2 Thermal detection threshold

The thermal detection threshold (TDT) is the lowest temperature difference that can be perceived during an increase or decrease from the baseline temperature (Kappers and Plaisier, 2019). Studies show that the TDT depends on at least four parameters (Lee et al., 1998; Jones and Berris, 2002). The four parameters are:

1. The temperature of the skin stimulated.

2. The rate of change of temperature.

3. The area of the skin stimulated.

4. The site of the skin stimulated.

**Figure 2:**
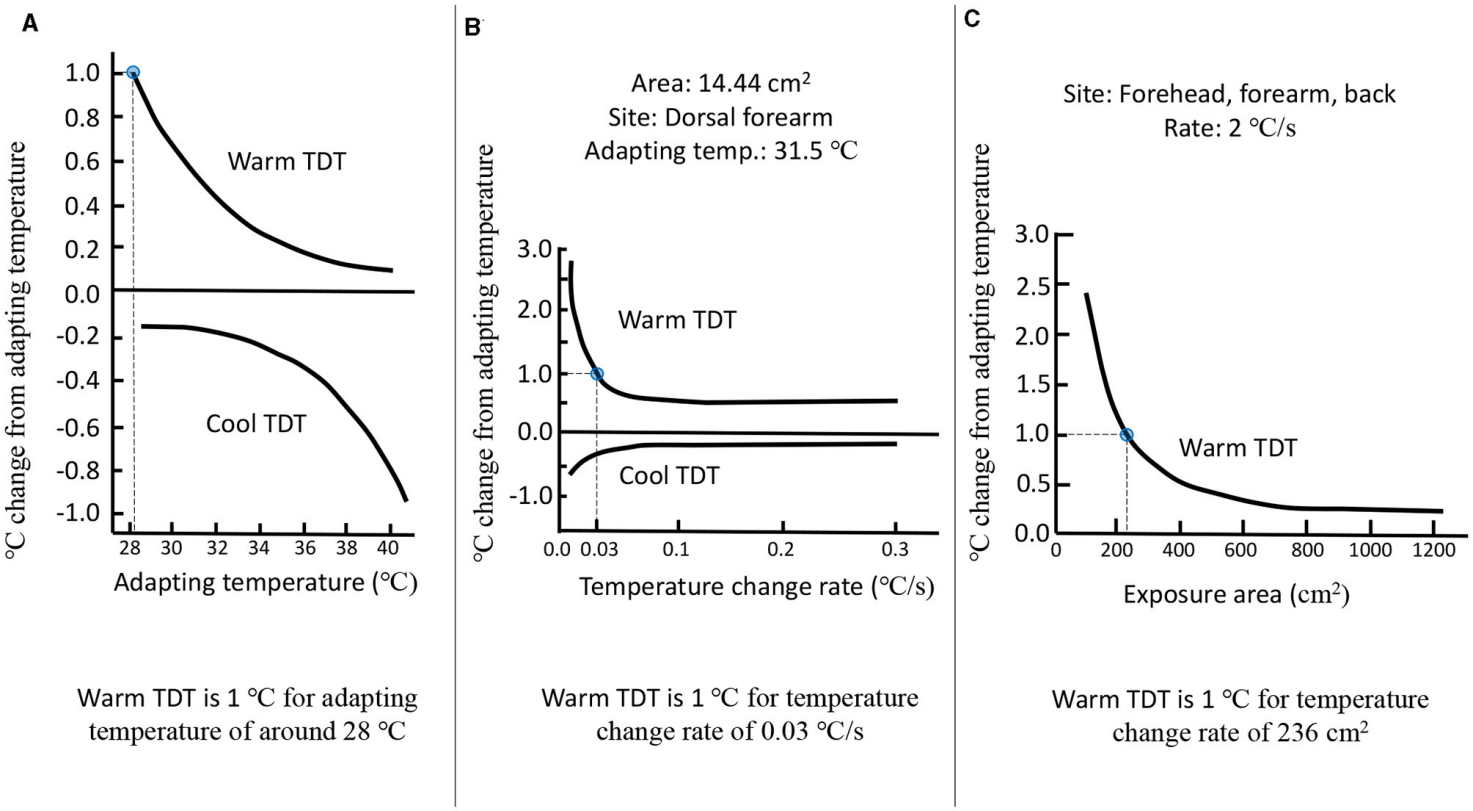
**(A)** Thermal detection threshold as a function of adapting skin temperature. **(B)** Thermal detection threshold as a function of rate of change of temperature. **(C)** Thermal detection threshold as a function of an area of the thermal stimulation. Adapted and redrawn from Lee et al. (1998).

With the increase in the adapted skin temperature, the TDT for warm sensation decreases, whereas the TDT for cool sensation increases (Figure 2A). At a skin temperature of 34°C, the warm TDT was measured to be 0.3°C, while the cool TDT was found to be 0.2°C. These measurements were obtained using a thermal stimulus of a certain area and a controlled rate of temperature change (Lee et al., 1998). For a fixed skin temperature, the thermal TDT depends on the rate of temperature change of the stimulus (Figure 2B). A higher rate of temperature change results in a lower warm TDT and a higher cool TDT. If the temperature is within the neutral range (30–36°C), then an increase of 5–6°C goes unnoticed by human subjects at a rate of 0.5°C/min. On the other hand, even a small increase in temperature can be detected at a much faster rate of 0.1°C/s (Kenshalo et al., 1968; Kenshalo, 1978). The area of the skin that is thermally stimulated also influences the TDT for thermosensation (Figure 2C). As the skin exposure area increases, the warm TDT decreases, whereas the cool TDT increases (Kenshalo et al., 1967; Stevens and Marks, 1971). Jamal et al. (1985) observed that the warm and cold thresholds were much higher for the ankle compared to the wrist, forearm, or thigh. In another study of thermosensitivity, 49 sites on the hand and 54 on the foot were mapped, and it was found that different regions in the foot can vary in thermosensitivity by a factor of five. Also, glabrous skin was found to be less thermosensitive than hairy skin, which is the opposite for tactile and pain sensitivity (Filingeri et al., 2018). Moreover, thermosensitivity depends on the climate of the place where the person is from (Lee et al., 2010). Inhabitants of tropical climate zones have a reduced sensitivity to detecting warmth. Thermal sensitivity is also influenced by age. The thermal detection thresholds for cold and warm were investigated by Stevens and Choo (1998) in a study with a sample of 60 persons ranging in age from 18 to 88 years. The study revealed a decrease in thermal sensitivity with advancing age, particularly in the peripheral regions, such as the foot.

Several psychophysical studies focused on how the knowledge of thermal detection can be applied to practical experiences. A research investigation was undertaken to examine temperature detection in various regions of the human body where a portable electronic device could potentially be held, including the thenar, finger, forearm, and upper arm (Wilson et al., 2011). Results showed that it was easier to detect the temperature change when the thermal intensity (the increase or decrease in temperature from the baseline) was higher. Moreover, cooling was detected much faster than warming (Wilson et al., 2011). In another study by Halvey et al. (2011), they implemented different types of textiles (nylon and cotton) to investigate the perceptual effect when they were placed between the skin (of the thenar, thigh, and waist) and a thermal stimulus. Participants reported a more comfortable sensation when textiles were used between the stimulus and their skin. The detection performance improved for thermal stimuli of higher intensity and a higher rate of change of temperature. Thermal detection also depended on the type of the textile, and it performed better for nylon than for cotton (Halvey et al., 2011). Ketna and Leelanupab (2017) conducted experiments to assess the perceptual efficacy of different types of stimulation (auditory, vibratory, and thermal) in a challenging environment characterized by high levels of noise and vibration. The study revealed that thermal stimulation exhibited the highest level of detectability in a noisy and bumpy environment. Furthermore, the outcomes were notably enhanced when multimodal stimulation was employed (Ketna and Leelanupab, 2017).

### 4.3 Thermal adaptation

Thermal adaptation is the ability of the thermoreceptors beneath the skin to adjust to a temperature stimulus. It is the gradual reduction of responsiveness to thermal stimuli over time (Kenshalo and Scott, 1966). When hands are dipped in warm water, the sensation of warmth gradually fades away, and after a while, the same water at the same temperature might feel cold (as observed by John Locke in the 17th century). Adaptation can manipulate the “physiological zero” of a person by shifting it to a lower or higher temperature (Stevens, 2013). Kenshalo and Scott (1966) conducted a study in which the participants were asked to sit in an air-conditioned room for 20 min followed by a task in which the participants had to adjust the temperature of a Peltier element placed on their forearm so that it was just detected as warm or cold. Several such trials were conducted over a period of 40 min during which the difference between the detection temperatures for warm and cold was noted. During this time period, there was an apparent rise in the difference between detection temperatures, which ranged from ~1°C to around 4°C. The reason behind this increase was thermal adaptation (Kenshalo and Scott, 1966).

### 4.4 Thermal spatial summation

The term “spatial summation” refers to the increase in perceptual or neurophysiological response when there is an increase in the area of stimulation (Stevens, 2013). It has been observed that thermal thresholds remain constant if the area of stimulus is doubled and the intensity of stimulus is halved (Jones, 2009). However, this phenomenon breakdown is usually near the pain threshold (Paricio-Montesinos et al., 2020). Studies were conducted to investigate spatial summation in different body regions (upper arm, forearm, back, chest, shoulder, and calf). It was observed that the perceived temperature was a power function of the intensity of the stimulus. The power function's exponent depends on the size of the area being stimulated and is smaller for larger areas (Marks, 1971; Stevens and Marks, 1971; Stevens et al., 1974). In another study, it was shown that thermal spatial summation can also happen for non-adjacent areas (areas that are far from each other on the body) of stimulation. In fact, thermal spatial summation can happen over the two arms by stimulating either one forearm or both forearms simultaneously (Rózsa and Kenshalo, 1977).

### 4.5 Thermal localization

Psychophysical experiments were conducted to investigate the capacity for localizing thermal perception. In a research study, Taus et al. (1975) administered radiant thermal stimuli to the participants' forearms and instructed them to discern the relative proximity of the radiant area to either the wrist or the elbow. The study findings indicate that there was a noticeable decline in performance when the thermal stimulus was of reduced intensity and positioned in closer proximity to the mid-line, which is the midpoint between the wrist and elbow. Conversely, the performance exhibited enhancement as the intensity increased and the distance from the mid-line increased (Taus et al., 1975).

## 5 Classification of thermal illusions

**Figure 3:**
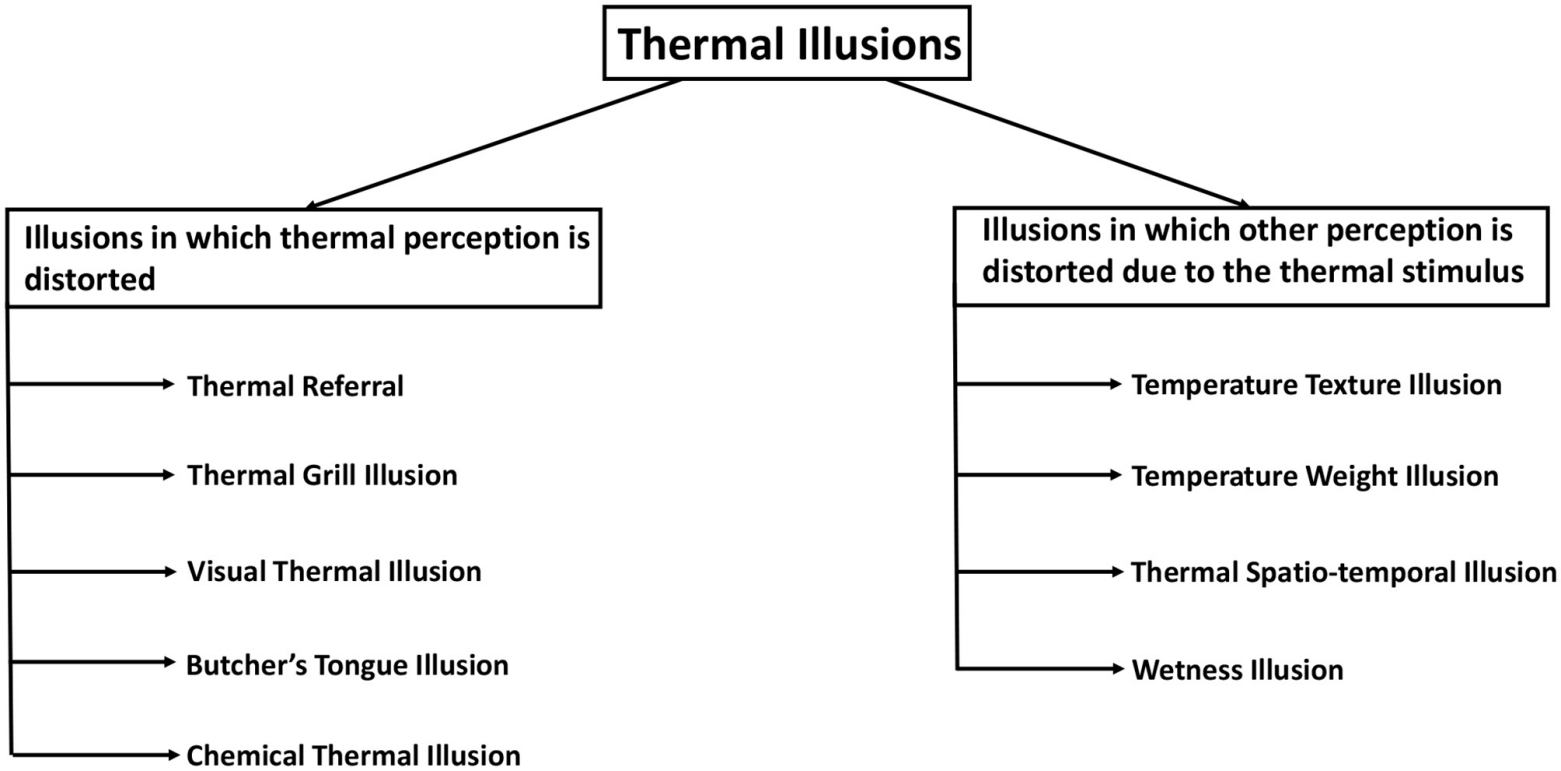
Classification of thermal illusions based on the type of sensation getting distorted. The illusions on the left are those in which thermal perception is influenced, whereas the illusions on the right are those in which the thermal stimulus influences another perceptual modality.

As sensory modality research progressed, more and more studies uncovered intriguing temperature perception phenomena in humans. Many of these phenomena present an illusory perception and can be called thermal illusions. An attempt to classify these illusions has been made in this section. Such categorization can aid in identifying the similarities and differences in the nature and mechanisms of thermal illusions. To introduce the breadth of thermal illusions two categories are considered (Figure 3):

1. Illusions in which the thermal perception is distorted.

2. Illusions in which the thermal stimulus distorts other sensory perceptions.

In the first category of thermal illusions, there is a discrepancy between the thermal perception experienced by the subject and the actual thermal stimulus that is presented to the subject. For instance, in Thermal Referral, a person can feel a thermal sensation in a region of the body where no thermal stimulus is present (Green, 1977b). In another illusion of the first category, an intense thermal perception can result from a considerably less intense thermal stimulus. The thermal grill illusion causes a person to experience a burning pain due to interlaced cold and warm stimuli that are individually not painful (Thunberg, 1896). Also, the thermal perception of a person can be manipulated by a stimulus to the other sense modalities. In the context of visual-thermal illusions, it is observed that the visual attributes of an object, such as its color, exert an influence on the corresponding thermal information of the object (Ho et al., 2014). Another similar example is the Butcher's tongue illusion, in which a dummy tongue is made to feel like someone's own using touch sensations, and then a visual-thermal stimulus on that tongue is felt in the real tongue by the subjects (Michel et al., 2014). Furthermore, the phenomenon of Chemical Thermal Illusion enables individuals to perceive sensations of warmth or coldness even in the absence of any actual thermal stimuli (Brooks et al., 2020).

The second category encompasses a collection of illusions wherein the thermal stimulus induces a discrepancy in another sensory modality. In the Temperature texture illusion, the skin temperature causes an alteration of the roughness perception of an object (Green et al., 1979). The perception of weight or force is distorted by the Temperature weight illusion (Weber, 1846). The perception of wetness is induced using a cold dry stimulus in Wetness illusion (Shibahara and Sato, 2016). In fact, temperature perception can also get intertwined with time perception or the ability to localize a stimulus. In the Thermal spatio-temporal illusion, the spatial and temporal properties of thermal stimulus cause distortion in the localization of the stimulus (Trojan et al., 2006; Singhal and Jones, 2016b). Furthermore, thermal illusions have been categorized in different ways with the aim of developing a thermal illusion taxonomy.

**Figure 4:**
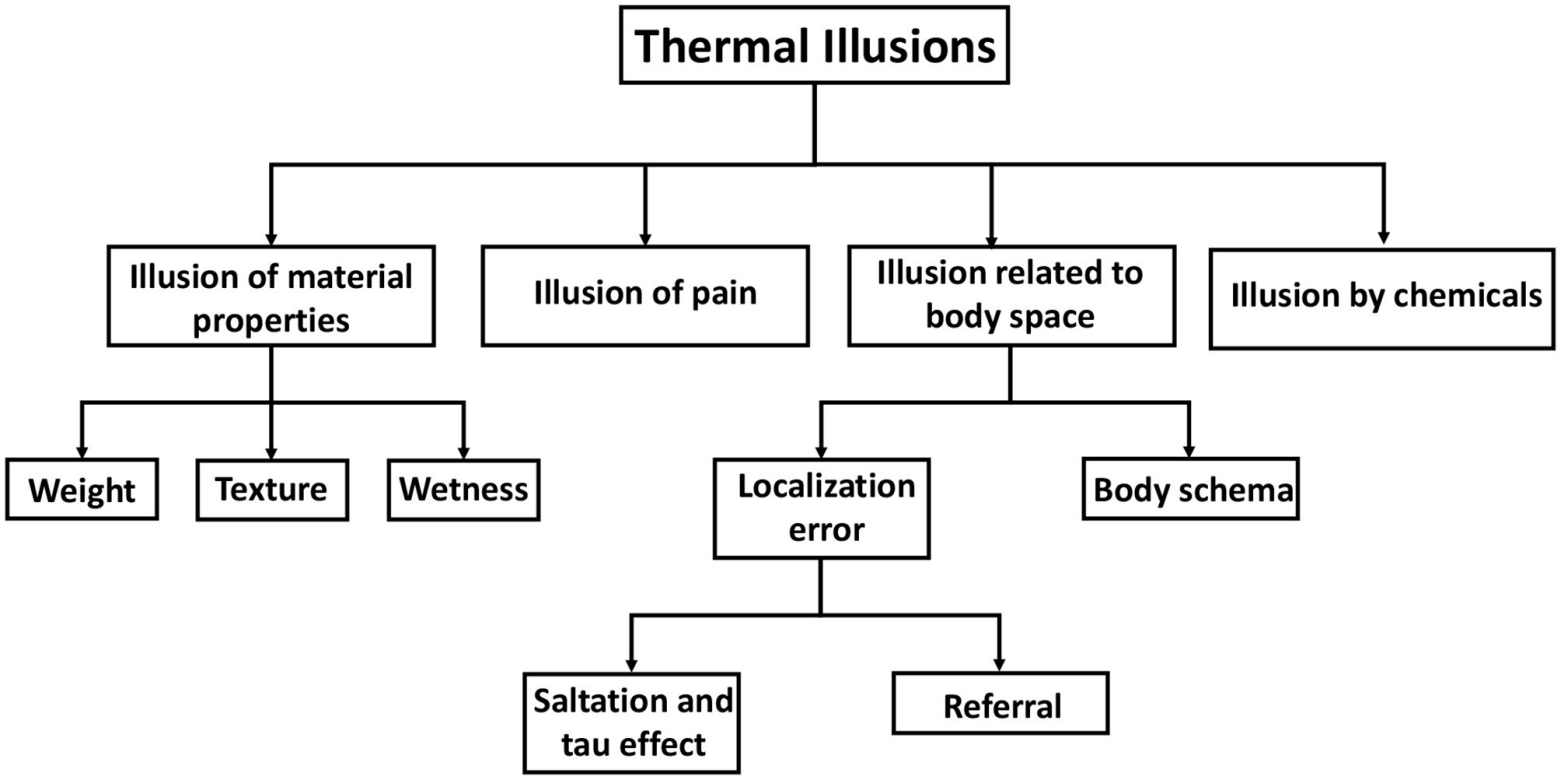
Taxonomy of thermal illusion. Thermal illusions are broadly classified into four types: illusions of material properties, illusions of pain, illusions by chemicals, and illusions related to body space. Material properties include the weight and texture of an object that is touched. Body space illusions include illusions related to body schema as well as errors in localizing thermal stimulus.

The classification of tactile illusions has been done by Lederman and Jones (2011), as summarized and taxonomically represented by Patel et al. (2019). The taxonomy for tactile illusions has served as a source of motivation and reference for the development of our taxonomy for thermal illusions. Thermal illusions are grouped into four categories or groups (Figure 4). One group involves those thermal illusions which influence the material properties of an object. Temperature-weight illusion, temperature-texture illusion, and wetness illusion fall under this category. The thermal-grill illusion is, however, kept in a separate group named “Illusion of pain” since it is associated with a noxious perception of temperature. The third group constitutes those thermal illusions that are related to the perception of body space by the individual. For instance, in the thermal spatio-temporal illusion, the ability of the subject to localize thermal sensations is distorted. Similarly, in thermal referral, a thermal sensation is perceived in a location where no stimulus is present. Again, the visual-thermal illusion and the Butcher's tongue illusion involve body ownership of an out-of-body object. Hence, they are classified under “Body Schema”. The chemical thermal illusion belongs within the fourth group, specifically labeled as “Illusion by chemicals.” A thorough review of each illusion and its corresponding psychophysical research has been undertaken. We have also endeavored to explore the theoretical foundations underlying some illusions. The objective of these deliberations is to obtain a comprehensive understanding of the research conducted on thermal illusions, with the aim of devising strategies for their application in thermal displays.

## 6 Illusion of material properties

The temperature stimulus provided to the skin impacts the perception of an object's material properties (Lederman and Jones, 2011). The illusions in this section can be used as a source of ideas for making haptic devices that show material properties.

### 6.1 Weight

Perception of the weight of an object can be influenced by many properties which are not normally considered to be the sensory information signaling weight. These properties include shape, volume, surface texture, density, and temperature (Jones, 1986). However, since in this review we are only focusing on thermal illusions, discussion of properties other than temperature is beyond the scope.

#### 6.1.1 Temperature weight illusion

**Figure 5:**
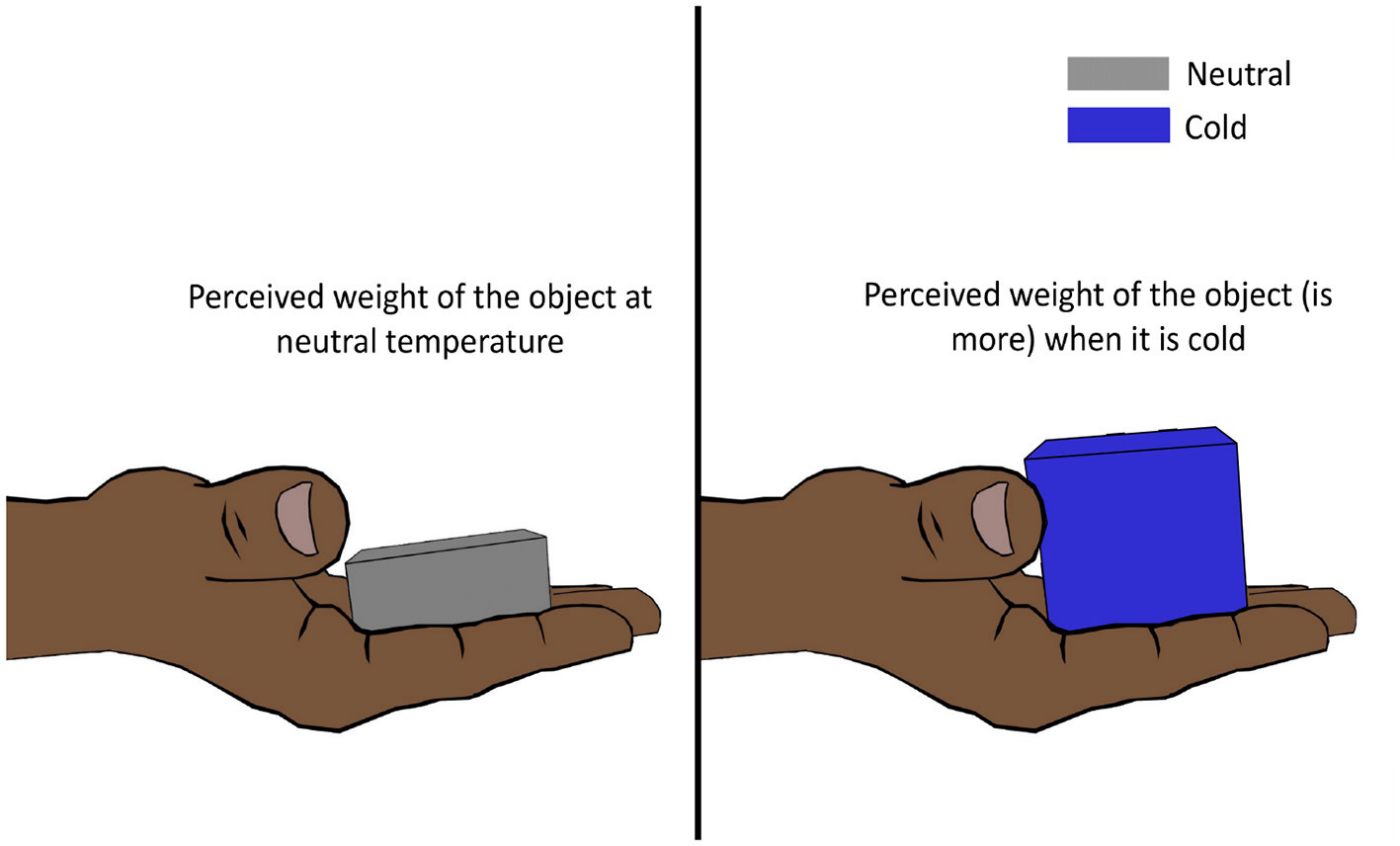
Temperature weight illusion. Lowering the temperature of an object increases its perceived weight. For an object at a higher temperature, the illusion is not perceived as strongly.

Weber (1846) discovered that placing a cold German dollar (thaler) on the forehead feels almost as heavy as placing two warmer identical dollars on top of the other on the forehead. This led him to conclude that cooling strengthens and warming lightens the pressure sensation on the skin. This then came to be known as Weber's phenomenon, the silver thaler illusion, or more commonly, the temperature weight illusion (TWI) (Weber, 1846; Stevens and Green, 1978; Weber and Ross, 1978). However, later, this theory was proved to be only partially correct by Stevens and Hooper (1982). Stevens and Hooper (1982) hired subjects to estimate the perceived heaviness of metallic stimulators of different masses and temperatures (cold, warm, and neutral), and they found that cold objects (25°C) felt significantly heavier (Figure 5), while warm objects (38°C) felt slightly heavier than neutral objects (33°C). The illusion was shown to be evident in different body regions (hand, arm, abdomen, back, and thigh) as well. Where cold-intensified heaviness was perceived across multiple body sites, warm-intensified heaviness was perceived in some regions (e.g., forearm) and not in others (e.g., forehead) (Stevens, 1979).

Controlled force and temperature stimuli were employed in a more recent study to re-evaluate the phenomenon and better comprehend the relationship between the tactile and thermal systems (Dunn et al., 2017). The study involved the application of two types of stimuli: a cooled tactile stimulus, where force was exerted together with a cooling stimulus that reduced the temperature from 32 to 28°C, and a tactile-only stimulus, where force was delivered at a thermally neutral temperature of 32°C. Almost all the participants reported the cooled tactile stimulus to be heavier than the tactile-only stimulus, but the effect is greater when the cooled tactile stimulus is applied after the tactile-only stimulus. In a subsequent experiment, the myelinated fibers of the ulnar nerve of the subjects were blocked (known as compression block) such that there was a loss of vibration (*Aβ* fibers blocked) and cold sensibility (*Aδ* fibers blocked), followed by a test where the same load was applied in combination with the cold and neutral temperature (Dunn et al., 2017). In the compression block condition, there was a significant reduction in the incidence of the TWI phenomenon. It was concluded that the TWI is more robust when all the nerve fibers are intact and that both the myelinated and unmyelinated fibers contribute to the phenomenon (Dunn et al., 2017).

Studies were also conducted to investigate the relationship between temperature and actively generated forces. Galie and Jones (2010) conducted an experiment to test whether the temperature of a surface has any effect on the perceived magnitude of the force applied by the finger on that surface. Results showed no such relationship between temperature and the perceived magnitude of actively generated force (Galie and Jones, 2010). However, in a recent study by Kuhtz-Buschbeck and Hagenkamp (2020), the effect of the TWI was observed in a grip-lift experiment where the subject lifted the test objects at varying temperatures (cold, neutral, and warm) from the palm of the non-dominant hand using the dominant hand. Recordings of the grip and lift forces revealed that the subjects applied more force (10% higher) when lifting a cold (18°C), heavy (700 g) object compared to a neutral object of the same weight (Kuhtz-Buschbeck and Hagenkamp, 2020).

Interestingly, the temperature of a surface in contact with the skin can generate the illusion of a push or pull. In a study by Watanabe and Kajimoto (2015), the participants were asked if they perceived any force on touching temperature-changing thermal elements. They reported an illusory sensation of pressure or push when the temperature of the display increased rapidly (27–37°C). Conversely, a pulling sensation was reported with a fast temperature drop (37–27°C) of the thermal display (Watanabe and Kajimoto, 2015). However, these psychophysical investigations are inadequate to describe the illusion's mechanism. The role of mechanoreceptors during temperature change can help explain the occurrence of TWI.

There are many slow-adapting (SA) mechanoreceptors that respond to both skin deformation and skin cooling and hence can be responsible for the TWI (Johnson et al., 1973). It is hypothesized that the activation of such receptive afferent fibers when holding a cold object results in the thermal intensification effect, causing the TWI (Lederman and Jones, 2011).

### 6.2 Texture

Skin temperature has been found in several studies to affect how an object's texture is perceived (Green, 1977a; Bolanowski et al., 1988; Zhang et al., 2009; Jones and Singhal, 2018). Understanding the relationship between skin temperature and texture perception has become more essential with the introduction of multi-dimensional tactile displays.

#### 6.2.1 Temperature texture illusion

**Figure 6:**
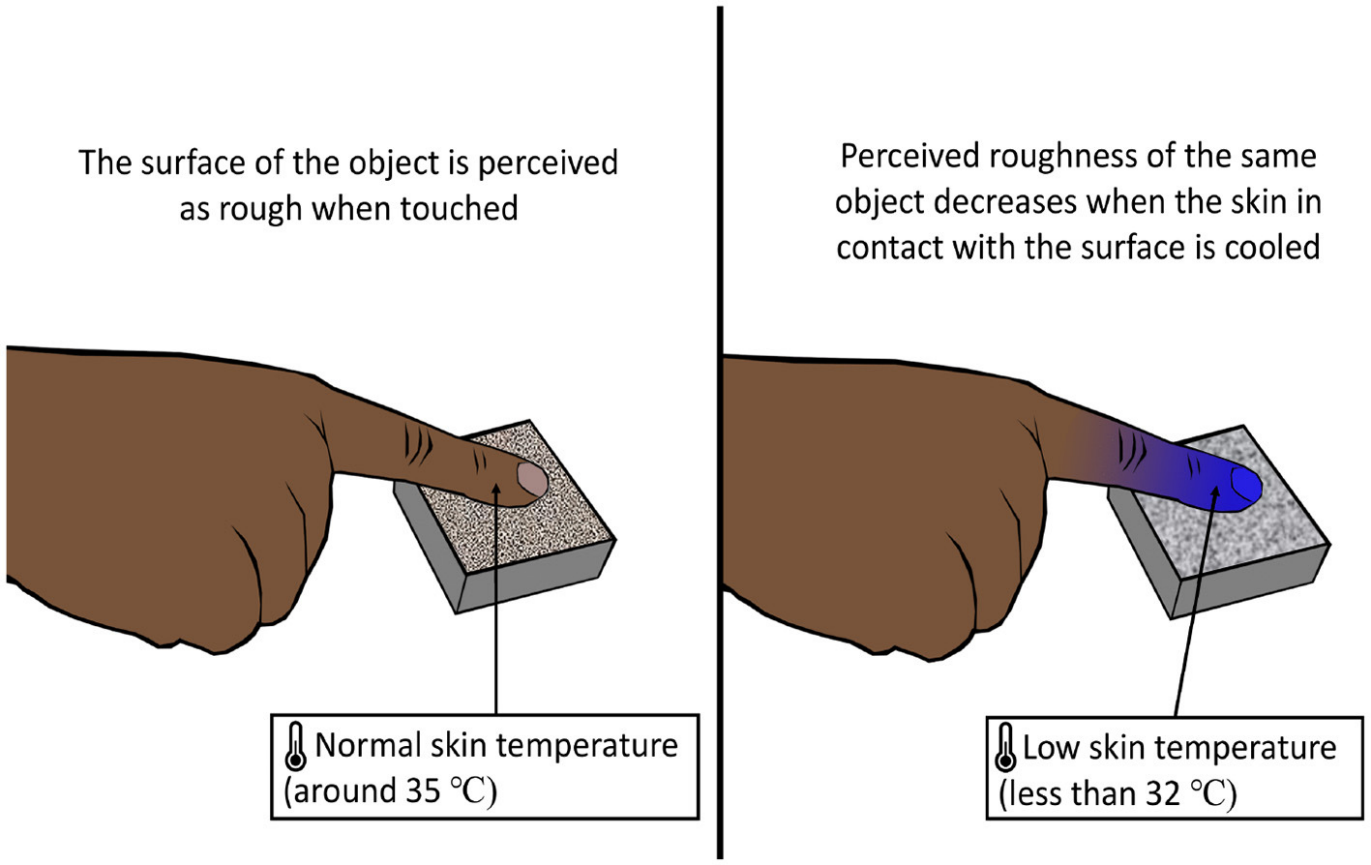
Temperature texture illusion. Lowering the skin's temperature reduces the skin's ability to perceive a rough surface, influencing texture perception.

The temperature of the skin has an effect on the roughness perception of an object in contact with the skin (Green et al., 1979). The apparent roughness declined as the skin temperature fell below 32°C (Figure 6). On the contrary, apparent roughness tended to increase with the increase in skin temperature although the effect was more dominant for cooling (Green et al., 1979). The phenomenon of alteration of texture perception of the surface of an object caused by a change in skin temperature can be called the temperature texture illusion (TTI).

Temperature also affects the vibrotactile sensitivity of the skin. The sensitivity of the skin to vibratory stimuli of varying frequencies decreased when warmed or cooled. Cooling, on the other hand, had a greater effect than warming on reducing sensitivity (Weitz, 1941; Green, 1977a; Bolanowski and Verrillo, 1982). Hence, similar to the temperature weight illusion, the TTI is also a result of the dependence of mechanoreceptors on temperature.

### 6.3 Wetness

Research suggests the perception of wetness is achieved by the integration of both tactile and thermal cues, as there are no specialized receptors that are dedicated to the sensation of wetness (Clark and Edholm, 1985; Filingeri et al., 2014b; Filingeri and Ackerley, 2017). The significance of the feeling of wetness lies in its capacity to enable humans to mitigate potential health hazards by avoiding unfavorable environmental conditions. For instance, the discomfort experienced when wearing damp clothing serves as a signal to dispose of the wet garment and prevent getting sick (Chau et al., 2018). This section explores the phenomenon of illusory perception of wetness.

#### 6.3.1 Wetness illusion

The wetness illusion refers to a phenomenon in which the perception of wetness is erroneously inferred by an individual due to the combined influence of thermal and tactile inputs. The sense of wetness is more sensitive to dynamic touch compared to static touch (Bergmann et al., 2012; Filingeri et al., 2014b). Dynamic touch refers to a tactile interaction when a contact surface undergoes relative movement over the surface of an object. In contrast, static touch involves a stationary contact area that does not exhibit any movement. The study revealed that a decrease in temperature is associated with an increase in the perception of wetness during static touch. However, in the case of dynamic touch, a decrease in temperature did not result in an increase in the perception of wetness (Filingeri et al., 2014b). The study conducted by Shibahara and Sato (2019) yielded similar findings, indicating that the illusion of wetness is more effectively induced through static touch rather than dynamic touch.

Wetness perception can be evoked using a dry and cold stimulus (Daanen, 2009; Bergmann Tiest et al., 2012; Filingeri et al., 2013, 2014a,c). Shibahara and Sato (2016) augmented the sensation of a wet cloth by controlling the temperature and softness of a dry cloth. The experiment demonstrated that the perception of wetness in a cloth can be replicated by manipulating two factors: reducing the temperature and enhancing the material's softness or compressibility. In a recent study conducted by Han et al. (2020), a prototype called Mouill was developed with the aim of inducing the illusion of wetness on the user's fingers during the manipulation of hard and soft items. A demonstration was given of a virtual reality application utilizing the device, showcasing the simulation of various things such as an ice cube, damp sponge, and chilled cola bottle.

## 7 Illusion of pain

This section is dedicated to the illusory perception of thermal pain. Currently, the thermal grill illusion is the only known phenomenon to induce the noxious perception of temperature using innocuous temperature stimuli.

### 7.1 Thermal grill illusion

**Figure 7:**
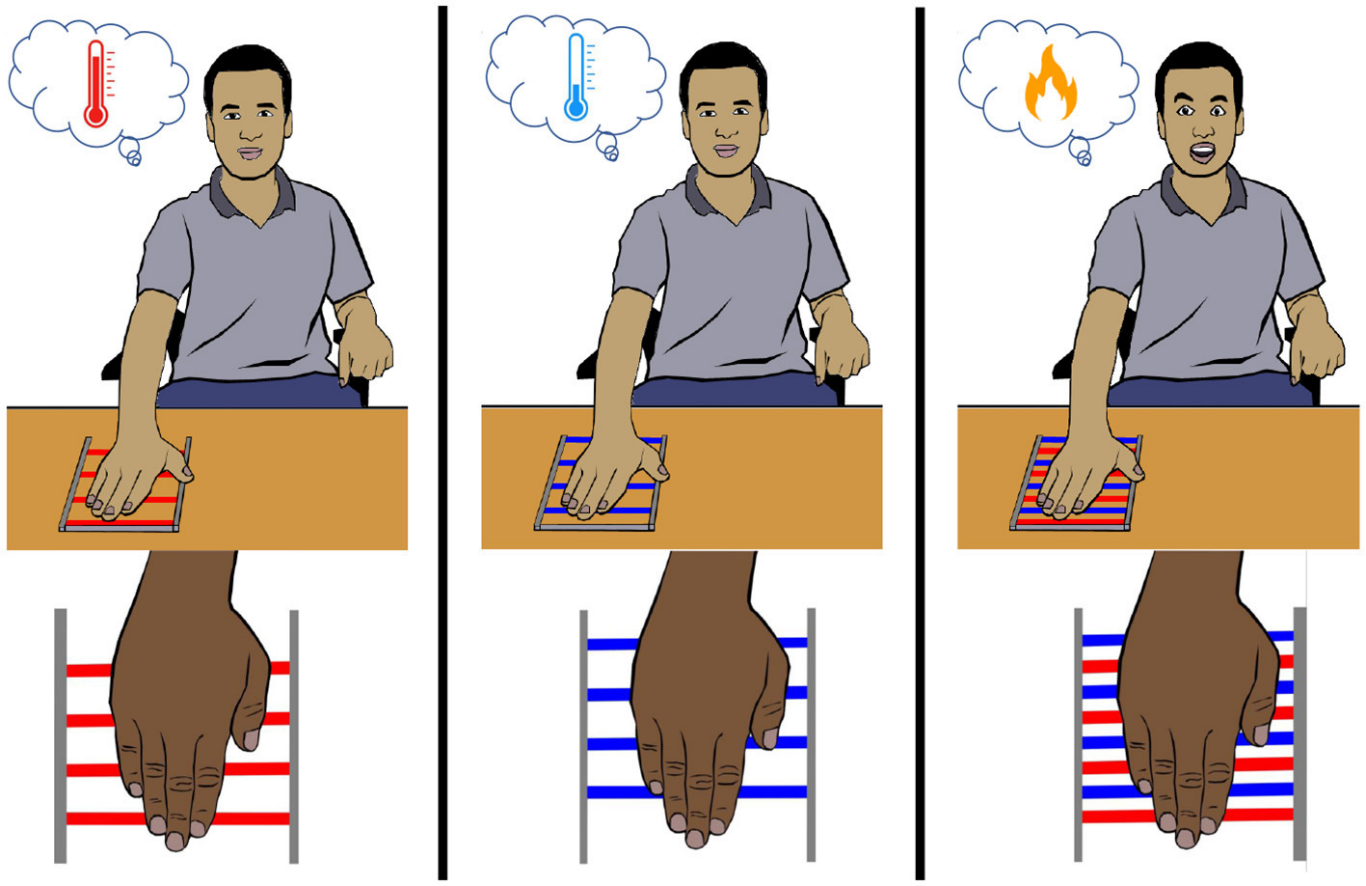
Thermal grill illusion. When an innocuous warm or cool stimulus is applied to the palm, a non-painful warmth or cold sensation is felt, respectively. However, when the same innocuous warm and cold stimuli are applied in an interlaced manner, a painful burning sensation is perceived.

In the 19th century, Thunberg (1896) and Alrutz (1898) independently discovered that interlaced warm and cold stimuli on the skin elicit a paradoxical sensation of burning pain. It was referred to as “synthetic heat” or the “thermal grill illusion” (Figure 7) (Fardo et al., 2020). Although the existence of the illusion was questioned by Jenkins (1938), influential research (Green, 1977a,b, 2002; Craig and Bushnell, 1994; Craig et al., 1998; Bouhassira et al., 2005) eradicated all the doubts, and thermal grill illusion (TGI) again became the object of study among researchers. The term “synthetic heat” was mostly used while investigating illusory heat without pain (Burnett and Dallenbach, 1928; Green, 1977b, 2002). On the other hand, the term “thermal grill illusion” denotes a thermo-nociceptive prickling sensation elicited by the grill of alternating warm and cold bars, known as the thermal grill. In studies, similar apparatus, such as alternating cold and warm spiral tubes (Thunberg, 1896; Bach et al., 2011) or alternating warm and cold thermodes (Defrin et al., 2002; Kammers et al., 2010; Marotta et al., 2015; Fardo et al., 2018), were used to elicit the illusory pain.

Though earlier studies showed that thermal grill illusion (TGI) can evoke a wide range of sensations (Burnett and Dallenbach, 1928), more recent studies suggest that TGI evokes mainly pain (Craig and Bushnell, 1994; Bouhassira et al., 2005; Kern et al., 2008a; Posada-Quintero et al., 2020). Several qualitative and quantitative approaches have been taken to understand the perception felt by the TGI. Subjects were asked to select from subjective descriptors of the illusion (e.g., pricking, cutting, stinging, burning, scalding, freezing, etc.) (Li et al., 2009; Bach et al., 2011), rate the illusion on verbal-numerical scales (e.g., verbally rate the pain between 0 and 100) (Scheuren et al., 2014) and visual analog scales (e.g., rate the pain on a visual scale of 100 mm) (Craig and Bushnell, 1994; Leung et al., 2005; Kern et al., 2008a,b; Adam et al., 2014), and also perform temperature matching tasks (Leung et al., 2005; Kammers et al., 2010; Marotta et al., 2015; Fardo et al., 2018; Ferrè et al., 2018). Factors such as genetics, gender, and psychological disorders were also found to impact the perceived intensity of TGI (Lindstedt et al., 2011; Averbeck et al., 2017). In the study by Averbeck et al. (2017), women reported a lower TGI-pain threshold than men. Patients with psychological disorders such as borderline personality disorder (BPD) or major depressive disorder (MDD) experienced less TGI intensity than healthy subjects (Boettger et al., 2013; Bekrater-Bodmann et al., 2015). Despite the fact that the neurophysiological mechanism underlying the phenomenon remains unknown, two significant theories have emerged.

“Addition or convergence theory” and “disinhibition or unmasking theory” are the two popular theories of TGI. The previous assumption in the late 19th century, known as the “addition theory,” was that concurrent warm and cold activation fuses into a sensation of heat. Craig and Bushnell (1994) proposed the “disinhibition theory,” according to which the TGI results from the disinhibition of C-polymodal nociceptors (heat-pinch-cold or HPC neurons) by the COOL neurons (i.e., neurons activated by mild cold temperatures) in the spinal cord (Craig and Bushnell, 1994; Craig et al., 1996, 1998). Both the theories are based on Johannes Müller's “law of specific nerve energies,” which postulates that for every sensory quality, there exists a distinct afferent pathway that receives signals from specific peripheral receptors and transmits them to the associated cortical targets in the brain, which finally determines the perceptual quality of the sensation (Norrsell et al., 1999; Fardo et al., 2020). Thus, for each sensation of heat, cold, or pain, there is a dedicated pathway or “labeled line” to transmit signals from the periphery to the brain.

The “addition theory” suggests that a polymodal pathway, activated by both warm and cold sensory inputs, encodes the linear summation of the inputs, resulting in the TGI (Green, 2002; Bouhassira et al., 2005). The greater the difference between the temperatures of the warm and cold sensory stimuli, the greater the perception of the synthetic heat caused by neural summation (Burnett and Dallenbach, 1927). Burnett and Dallenbach (1928) and Gritman and Dallenbach (1929) developed mathematical models based on the assumption that the inputs from both warm and cold temperatures activate polymodal pathways, and then the summation of the inputs causes increased firing rates, mimicking that of painful stimulation. The polymodal pathways are proposed to be second-order wide-dynamic range (WDR) neurons in the dorsal horn of the spinal cord (Green, 2002; Bouhassira et al., 2005). These WDR neurons, which respond to mechanical and thermal stimulation, are suggested to be involved in the painful perception of both noxious stimulation and that of thermal grill (Mendell, 1966; Maixner et al., 1986; Hao et al., 1992; Coghill et al., 1993; Khasabov et al., 2001). However, a more accepted theory of TGI is that of the “disinhibition theory.”

The “disinhibition theory” suggests that there are two separate steps involved in the painful perception of TGI (Craig and Bushnell, 1994; Craig et al., 1998; Craig, 2003). First, the warm stimulation causes the inhibition of COOL neurons in the dorsal horn of the spinal cord. The COOL neurons respond to temperatures below 27°C and are assumed to inhibit pain at the thalamo-cortical level. Second, due to the reduced inhibition of the COOL neurons, the pain-sensitive C-polymodal nociceptors (HPC neurons) get disinhibited. Hence, the “warm inhibits cold” mechanism results in the reduction of the “cold inhibits pain” mechanism, which finally leads to “disinhibition of pain.” These assumptions of the mechanism of “warm inhibiting cold” and “cold inhibiting pain” are based respectively on the electro-physiological studies conducted by Craig and Bushnell (1994) and the observations of nerve blocks altering cold, warm, and pain perception (Mackenzie et al., 1975; LaMotte and Thalhammer, 1982; Fruhstorfer, 1984; Wahren et al., 1989; Yarnitsky and Ochoa, 1990). A mathematical model of TGI based on the “disinhibition” theory has been proposed by Karmakar et al. (2023). Both “addition” and “disinhibition” theories are based on the recruitment of a specific type of neuron (WDR or HPC) for painful perception. Nevertheless, Fardo et al. (2020) recently proposed an alternative hypothesis based on the theories of population coding.

Population coding models are widely accepted in vision neuroscience and are based on the pattern theory of color vision and the across-fiber pattern approach (Erickson, 1973). The hypothesis proposed for TGI was inspired by the gate control theory (Melzack and Wall, 1962, 1965). It supported the view that perception relies upon spatio-temporal patterns of activity in the central nervous system, distributed across nociceptive and non-nociceptive pathways (Melzack and Wall, 1962, 1965). The main concept of population coding was that perception is probabilistically dependent on the neural response of a distinct population of neurons at different levels of neuraxis (Averbeck et al., 2006; Quiroga and Panzeri, 2009; Panzeri et al., 2015). Hence, Fardo et al. (2020) hypothesized TGI as a consequence of a certain neuronal pattern which is interpreted as a burning pain in the brain. The mechanism behind TGI has been a mystery for decades, and researchers are still trying to unravel it. Understanding TGI might also help researchers with studying chronic pain perception and pain management (Shin and Chang, 2021). Attempts to incorporate the TGI into thermal displays have been made (discussed in later sections).

## 8 Illusions related to body space

This section discusses illusions related to the spatial representation of the body. The category is again divided into two sub-categories, namely, localization error and body schema. A localization error is one in which there is a distortion in the perception of the stimulus location. Body schema is the awareness of the relative positions of distinct body parts in external space.

### 8.1 Localization error

The error in localization can happen either due to the non-uniformity of the stimuli as in Thermal Referral or due to the influence of other perceptual information as in Thermal Spatio-temporal Illusion. Each of the two illusions has been discussed below.

#### 8.1.1 Referral: thermal referral

**Figure 8:**
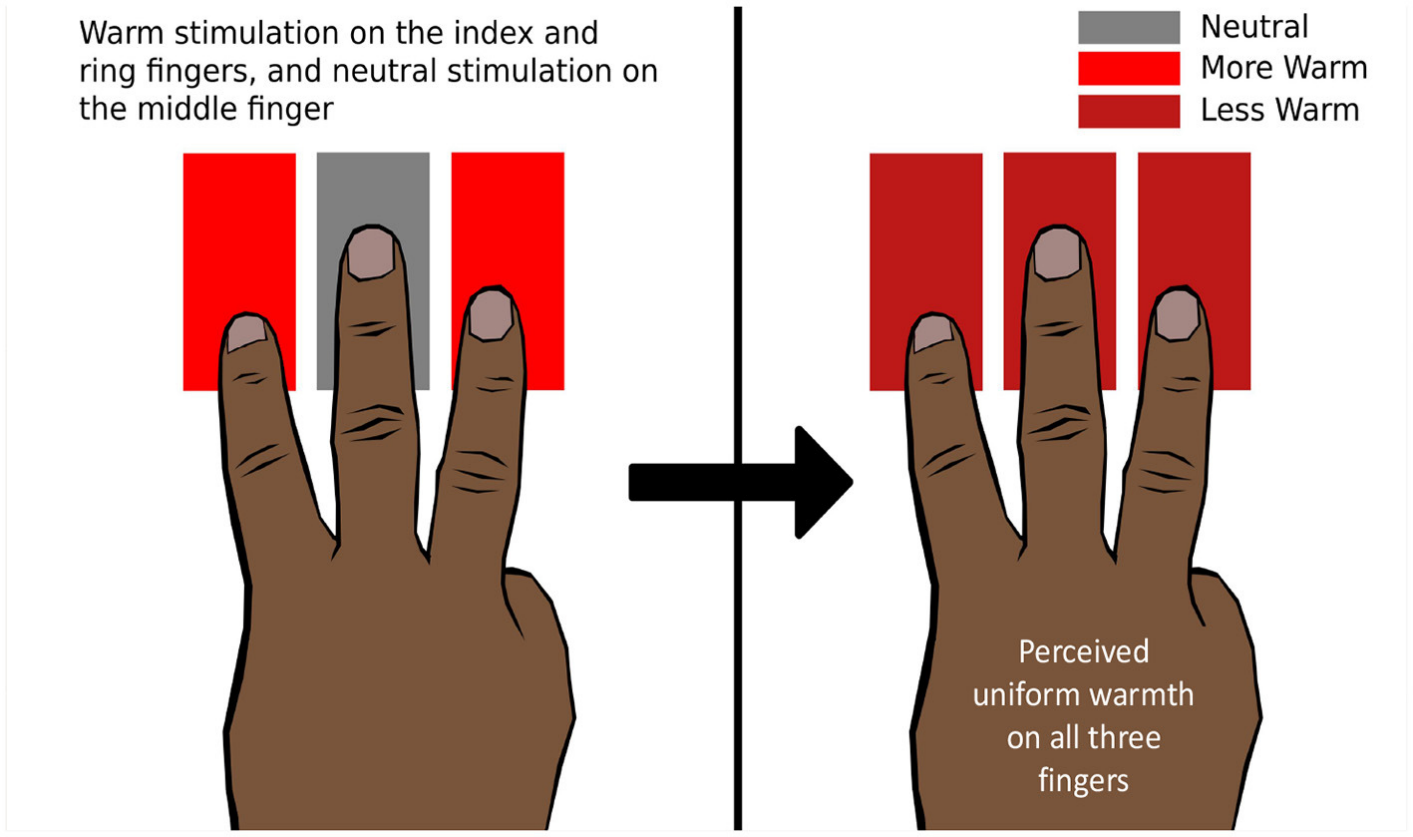
Thermal referral. The ring and index fingers are presented with a warm thermo-tactile stimulus, and the middle finger is presented with a neutral tactile stimulus. All three fingers perceive uniform warmth, which is less intense than the physical intensity of the warm stimulus.

Green (1977b) demonstrated that when the index and annular fingers are thermally stimulated by hot or cold temperatures while the middle finger is kept on a thermally neutral stimulator, the middle finger also perceives hot or cold temperatures, respectively. This illusion is called “thermal referral” (TR) since the thermal sensation at the outer fingers is referred to the middle finger (Figure 8). However, the illusion disappears when the middle finger is lifted or kept just above the stimulator (Green, 1977b). Hence, it is necessary to stimulate the middle finger with a tactile sensation for the phenomenon to occur. TR can also be elicited using two fingers by giving tactile stimulation to one and thermal-tactile stimulation to the other. TR was elicited in other areas of the body as well, including the forearm (Green, 1977b).

In a study by Ho et al. (2011), it was observed that the sensation of cold or hot is uniform across three fingers during TR, but the perceived intensity on the outer fingers is lower than the physical intensity applied to the outer fingers (Ho et al., 2011). It is proposed that the TR phenomenon is mediated by two stages. First, the thermal perceptions across multiple stimulated areas are homogenized to determine an overall intensity perception. In the second stage, this resulting intensity is referred to or localized to different regions of the body based on the tactile stimulation (Ho et al., 2011). Ho et al. (2010) also investigated how thermal and tactile modalities coordinate to process the localization information.

The strength of mislocalization in TR depends upon the somatotopic distance or the distance between the sites represented in the cortical topography. The strength of the illusion is reduced as the somatotopic distance increases, but the spatiotopic distance, or the distance between the sites on the body, has no effect (Ho et al., 2010). Watanabe et al. (2014) studied the mutual interaction between the thermal referral of two thermal-tactile stimuli on the forearm and yielded interesting results. The occurrence of TR from the elbow side to the wrist side was much more than that in the reverse direction. Hence, the sensation migrates better toward the periphery than toward the center (Watanabe et al., 2014). Further research observed the possibility of TR using radiant stimuli.

Cataldo et al. (2016) investigated whether TR is possible using purely thermal stimulation, i.e., the absence of any tactile object to which temperature can be attributed. An infrared light bulb was employed to deliver radiant thermal stimuli to the index and ring fingers, whereas no thermal stimulation was applied to the middle finger. The results concluded that TR is indeed possible using purely thermal stimuli and in the absence of any tactile stimulation. This raised questions about the basic mechanism underlying thermal referral, which is believed to be dominated by tactile information over thermal signals.

The phenomenon of the thermal sensation produced by the outer thermal stimuli being referred to as the central stimulation spot was termed as “domination” (Green, 1977b). However, a more recent study by Arai et al. (2021) challenged the “domination” hypothesis and introduced the concept of hot-cold confusion. In their first experiment, they presented three thermal stimuli in different patterns (alternate, adjacent, mixed, and same) of hot and cold to the forearm and observed that the participants sometimes perceived the inverse thermal sensation (cold perception of hot stimuli and vice-versa) of what was presented both at the center spot and the outer spots. Based on their findings, Arai et al. (2021) hypothesized that there is a mutual effect between the hot and cold stimuli such that sometimes the outer stimulation influences the central stimulation and sometimes the reverse happens, and it did not matter which stimulus was greater in number. Moreover, the location (proximal or distal) of the thermal stimulus influenced the mutual effect. When the center stimulus was warm, the outer thermal stimulus near the elbow was also perceived as warm. Conversely, the cold stimulus at the center tends to influence the outer thermal stimulus near the wrist. This finding was consistent with the study by Watanabe et al. (2014).

A uniformity of stimulation across fingers is necessary for the TR to occur. In Green's case, raising the middle finger produced non-uniformity in tactile stimulation, which resulted in a thermal perception of non-uniformity (Green, 1977b). In Cataldo's case, the tactile stimulation across the fingers was homogeneous, which appeared to be essential for the illusion. Hence, thermal referral requires all or none of the three fingers to be tactically stimulated (Cataldo et al., 2016). Thermal referral, according to Cataldo et al. (2016), can be explained by low-level spatial summation mechanisms in the thermoceptive sensation rather than a multisensory, cognitive process of object perception. Nevertheless, it is evident that more studies are required in order to paint a clear picture of the mechanism behind TR.

#### 8.1.2 Saltation and tau effect: thermal spatio-temporal illusion

**Figure 9:**
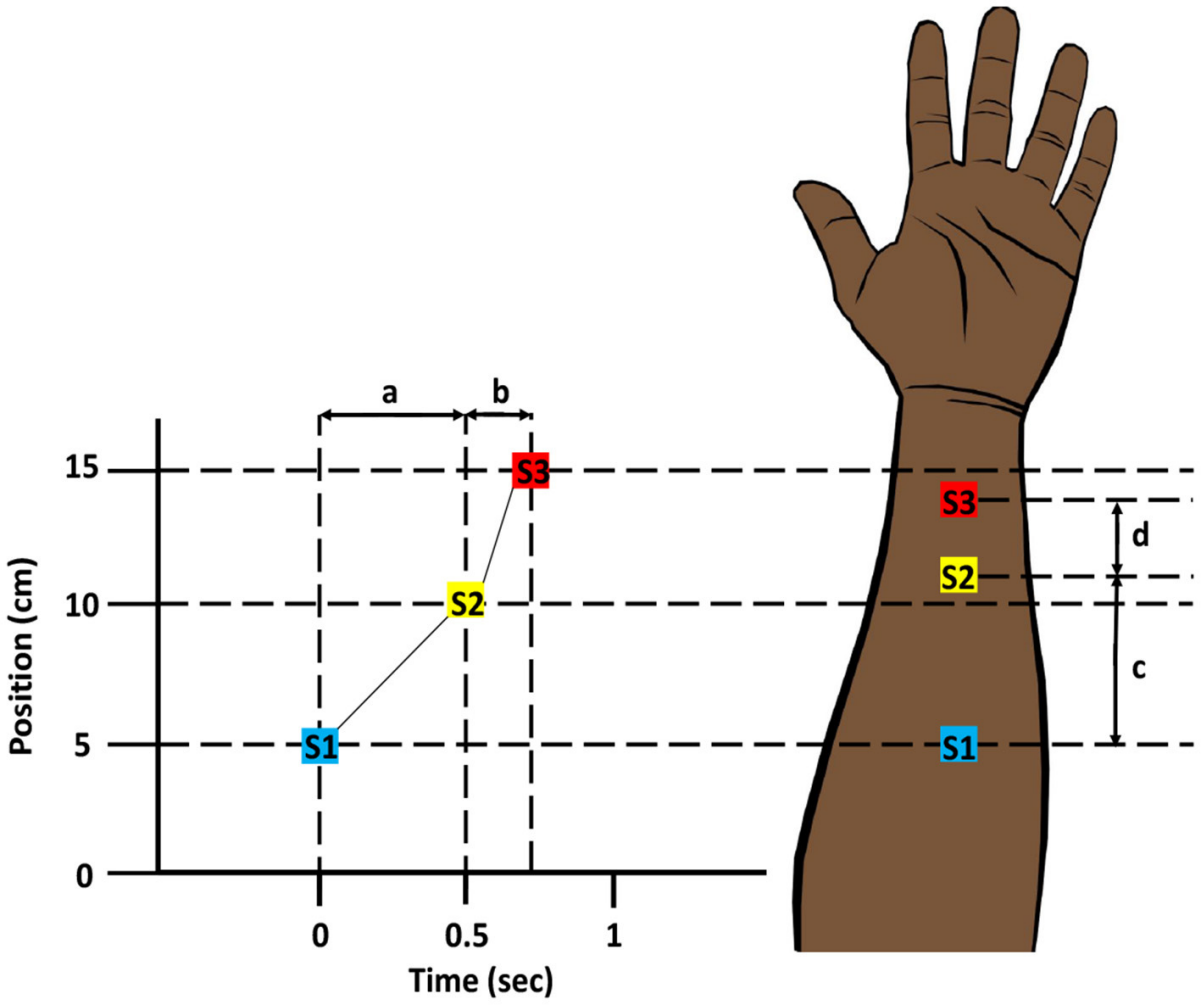
Thermal Spatio-temporal Illusion. Three thermal stimuli (S1, S2, and S3) are applied successively at three different positions on the skin. Since the time interval (b) between the second and third stimuli is shorter compared to the time interval (a) between the first and second stimuli, the perceived distance (d) between the second and third stimuli is less than the perceived distance (c) between the first and second stimuli. Modified and redrawn from Goldreich (2007).

Space-time illusions have been widely demonstrated for mechanical stimuli on the skin, but comparable illusions can also be created for thermal stimuli (Lederman and Jones, 2011). The “tau-effect” and later the “sensory saltation” are illusions in which the time delay between mechanical stimulations can alter the perception of space (Helson, 1930; Geldard and Sherrick, 1972). In 1930, Helson showed that when three spots on the back of the hand or arm are touched in quick succession, the perceived distance between the stimulation sites is determined by the time gap between them (Helson, 1930; Helson and King, 1931). Later, Geldard and Sherrick (1972) discovered a phenomenon in which successive mechanical stimuli applied to different locations across the skin appear to move progressively. This illusory occurrence was originally dubbed “the cutaneous rabbit,” after a rabbit's hopping movement, but eventually became known as the “sensory saltation” (saltation is Latin for jumping). In a simpler sense, the saltatory effect is the displacement of one stimulus toward another as a function of time (Geldard, 1982, 1985). Thermal stimulation can also be used to create this illusion and is known as “thermal sensory saltation” or the “thermal spatio-temporal illusion” (Figure 9) (Trojan et al., 2006; Singhal and Jones, 2016b).

Psychophysical studies were conducted to observe the spatio-temporal relationship using thermal stimuli of different types. Singhal and Jones applied four cooling (Singhal and Jones, 2016b) and four warming (Singhal and Jones, 2016a) pulses to the forearm in different spatial and temporal sequences using thermoelectric modules. After each trial, participants indicated where they thought the initial two pulses were located. When the time between the second and third pulses was short (0.2 s) and the distance between them was higher, the position of the second pulse shifted significantly in the direction of the third pulse. Hence, the time delay between heat stimulations might affect where they are perceived (Singhal and Jones, 2016b). In another study, Trojan et al. (2006) used CO_2_ laser to demonstrate the tau-effect in thermoreceptive and nociceptive pathways. Two test stimuli, followed by a reference stimulus, are presented to the dorsal forearm. The time delay between the test stimuli is varied between 60 and 516 ms, and a 3D tracker is used to indicate the perceived positions of the stimuli. The results showed a shift in the apparent position of the first test stimulus toward the second one. The shorter the time interval, the bigger the displacement (Trojan et al., 2006).

Goldreich (2007) proposed a Bayesian perceptual model to explain several tactile spatio-temporal illusions. It explains the contraction in perceptual length in the “tau-effect” or cutaneous rabbit by suggesting that the brain incorporates expectations for speed in order to overcome the limitations of spatial and temporal uncertainty inherent in the sensorineural signal. Thermal spatio-temporal illusion can be used in thermal displays for communication purposes, as discussed in the following sections.

### 8.2 Body schema

Illusions in this sub-category involve body ownership. These thermal illusions can be considered extensions of the haptic illusion related to body schema as described by Lederman and Jones (2011). Both the Visual Thermal Illusion and the Butcher's tongue illusion constitute some form of the rubber hand illusion (RHI). RHI refers to the misperception of a fake rubber hand as one's real hand (Botvinick and Cohen, 1998).

#### 8.2.1 Visual thermal illusion

Various colors are frequently connected with different temperature perceptions although some research disagrees. For example, the color red is often associated with warmth and the color blue with coolness. In fact, several researchers studied the popular Hue-Heat Hypothesis, which states that a light wave of dominant frequency closer to the blue end of the visual spectrum is perceived as cold and that toward the red end is perceived as warm (Candas and Dufour, 2005). However, a number of studies rejected this hypothesis and suggested that hue or color has no effect on thermal perception. For example, Mogensen and English (1926) showed that wrapping equally heated cylinders with papers of different colors has little effect on the thermal perception of those cylinders (Mogensen and English, 1926). Berry (1961) conducted a study where the room was illuminated with lights of different hues and the subjects were asked to judge the temperature of the room. Results showed an insignificant effect of hues on their temperature perception (Berry, 1961). However, there are many studies that suggest a correlation between visual information and thermal perception as well.

Research shows that visual information can often manipulate thermal perception in certain conditions. In a study by Ho et al. (2014), the implicit association test (IAT) and cross-modal priming task are used to observe the color-temperature correspondence (red-warm and blue-cold). The IAT is a test that assesses the degree to which two concepts are associated (Greenwald et al., 1998). The results revealed that reaction times are longer for in-congruent (red-cold and blue-hot) conditions than for congruent (red-hot and blue-cold) conditions (Ho et al., 2014).

**Figure 10:**
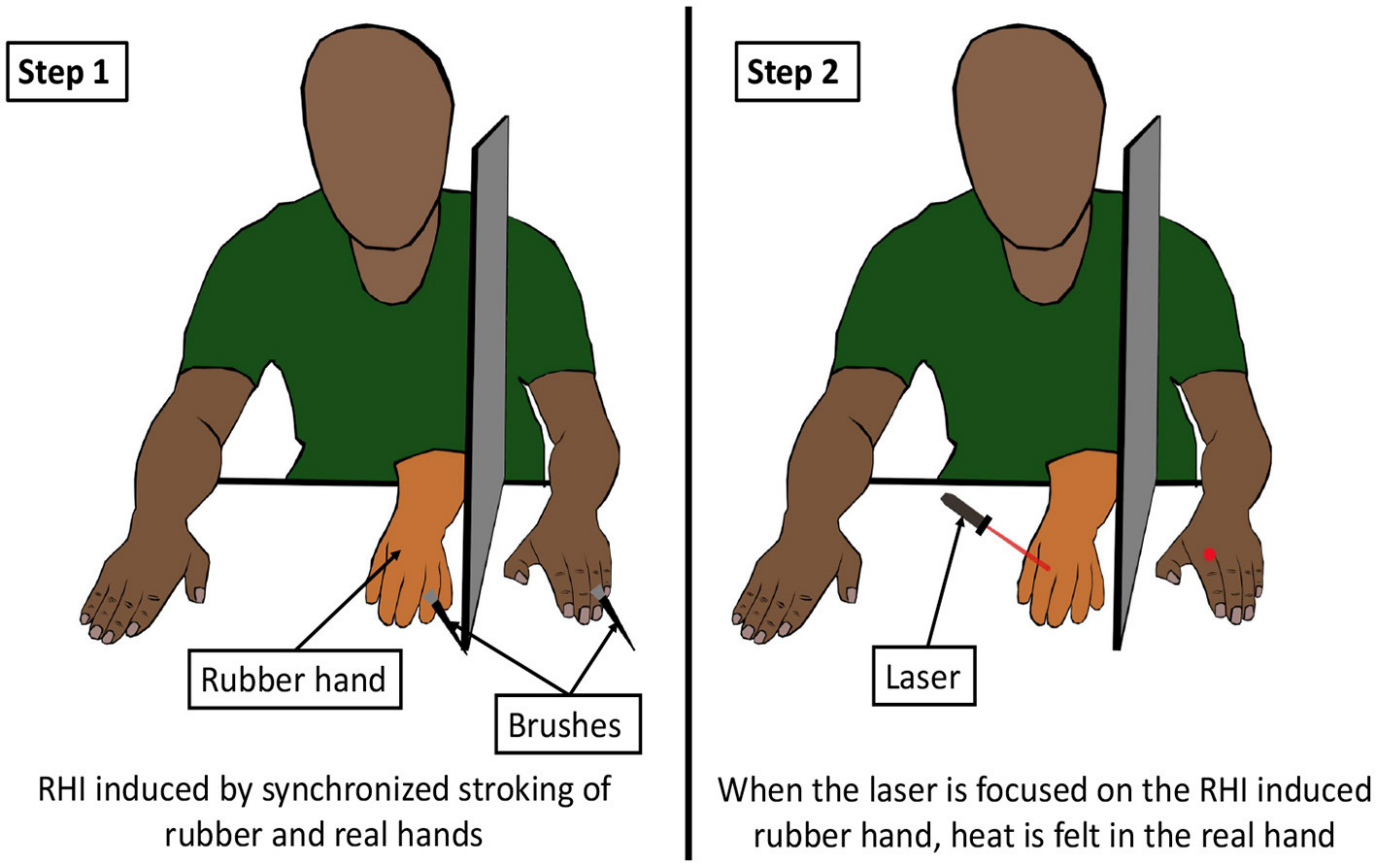
Visual thermal illusion. The first step of the illusion is to perform a body ownership experiment like the rubber hand illusion (RHI). RHI is induced by stroking the dummy hand and the real hand (hidden from the subject by a barrier) in a synchronous manner. After the subject perceives the rubber hand as their own hand, a thermal stimulus is presented on the rubber hand by a laser. This results in a thermal or tactile sensation in the real hand (Durgin et al., 2007).

The effect of vision on thermal perception has been studied in the rubber hand illusion as well. Kanaya et al. (2012) observed that the mere sight of ice being touched to a rubber hand, which was perceived as one's own hand due to rubber hand illusion (RHI), caused a cold sensation in the real hand. In RHI, a rubber hand, on stroking simultaneously with a real hand, causes the person to feel the rubber hand “as if it is his/her own hand” (Botvinick and Cohen, 1998). In another experiment, it was shown that an RHI-induced rubber hand caused the individual to experience thermal and tactile sensations when stroked by laser beams (Figure 10) (Durgin et al., 2007). In fact, the rubber hand illusion was itself induced using visual-thermal stimuli by Trojan et al. (2018). Thus, it is evident that the visual information of an object can influence the thermal perception of it. A visual-thermal illusion (VTI) can be defined as a phenomenon in which the visual appearance of an object affects the thermal perception of that object.

Various other psychophysical experiments were conducted to establish a relationship between visual and thermal perception. A pilot study showed how color exposure impacted thermal thresholds and thermal detection. Red light was shown to decrease the cold pain thresholds, while green light increased the hot pain thresholds and detection of warm stimuli (Landgrebe et al., 2008). Another study found an association between color cues and thermal pain threshold using virtual reality. Seeing a virtual red arm decreased the hot pain threshold on the right wrist compared to seeing a virtual blue arm (Martini et al., 2013). Interestingly, color cues were also shown to be associated with the thermal sensation of the nasal cavity. Sniffing a green solution was associated with a cooling sensation and a red solution with a warming sensation (Michael et al., 2010). However, in visual-touch interaction, it is difficult to tell which one of vision and touch is dominating the perception.

Multisensory studies suggest the dominance of vision over touch (Spence et al., 2001). However, Ernst and Banks (2002) proposed a Bayesian model which suggested that visual information might not always dominate haptic information. The model predicts the integration of visual and haptic information using a maximum-likelihood estimate (MLE).

#### 8.2.2 Butcher's tongue illusion

The Butcher's tongue illusion (BTI) is a novel variant of the rubber hand illusion (RHI) where it is extended to the tongue (Michel et al., 2014). As discussed earlier in this paper, in RHI, a rubber hand can be made to feel like someone's own hand (Botvinick and Cohen, 1998). Similarly, in BTI, the participant looks at the mirrored image of a rubber tongue while it is being stimulated synchronously with the real tongue. The participant reports ownership of the dummy tongue after the stimulation. The stimulation can be done either by touch or by a laser pointer. In the case of the laser, the participants reported a feeling of either tactile or thermal stimulation in their own tongues (Michel et al., 2014). In a paper, Spence (2015) suggests that in the future, the BTI may be used to take flavors out of the mouth.

From all of the discussions on thermal illusions, it is clear that there is an opportunity to create multimodal haptic displays that incorporate these illusions. In the next section, several thermal displays that have been developed are briefly discussed. Learning about the techniques used in these displays can lead to new ideas, allowing for better displays to be produced in the future with broader applications.

## 9 Illusion by chemicals

Certain chemicals are known to elicit a thermal perception of cold or warmth. This section discusses the illusory perception of temperature due to activation by chemical stimuli.

### 9.1 Chemical thermal illusion

Brooks et al. (2020) conducted a study on temperature illusions associated with the trigeminal nerve located in the nasal region. The trigeminal nerves can be activated by both temperature and chemicals to produce a sensation of cold or warmth. In the study, Brooks et al. (2020) developed a stand-alone device that has an atomizer to spray custom-made “thermal” scents in the user's nose. A device coupled to the virtual reality headset atomized capsaicin, or cayenne pepper tincture, to create the illusion of warmth, immersing the user in a virtual world where they are warmed by a furnace. Similarly, eukalyptol was atomized in the user's nose to mimic a cold sensation while virtually experiencing a gust of cold wind. Additional trigeminal scents employed in the study encompassed peppermint essential oil and linalool for cooling, while methyl salicylate and thymol were utilized for warming effects. Brooks et al. (2020) proposed trigeminal-based thermal illusions as a potential substitute for Peltier-based thermal displays, which are known to have significantly higher energy consumption.

As previously mentioned, a number of chemical substances have been discovered that elicit the activation of thermoreceptors, resulting in the perception of cold or warmth (Liljestrand and Magnus, 1922; Schreiner, 1936; Gollwitzer-Meier, 1937; Schmidt, 1949; Hensel, 1973). However, it can be contended whether these phenomena can truly be classified as illusions, as they do not involve the misinterpretation of external cues. Recent research findings have elucidated the mechanisms by which specific transient receptor potential (TRP) channels exhibit responsiveness to both heat and chemical stimuli (Schepers and Ringkamp, 2010). TRP channels are cationic channels that are involved in the mediation of a variety of sensations (Vriens et al., 2014). There are 28 different TRP channels in humans, and they can be grouped into six families. Among these families, only three families, namely, the vanilloid TRP channels (TRPV), the melastatin or long TRP channels (TRPM), and the ankyrin transmembrane protein channels (TRPA), are involved in the thermoreception of innocuous and noxious temperatures (Pedersen et al., 2005).

Different TRP channels are activated at different temperatures and also by different chemical agonists. The transient TRPM8 ion channel is activated at temperatures below 26°C, as well as by agents like menthol, eucalyptol, and icilin, which produce the cold sensation (McKemy et al., 2002; Peier et al., 2002; Chuang et al., 2004; Bautista et al., 2007). Noxious cold is transduced by TRPA1 ion channels, which can also be activated by icilin, mustard oil, cinnamaldehyde, allicin, or bradykinin (Story et al., 2003; Bandell et al., 2004; Jordt et al., 2004; Macpherson et al., 2005; Bautista et al., 2006; Story and Gereau, 2006). Temperatures above 33°C activate the TRPV3 ion channel, resulting in a warm sensation. Camphor is also known to activate the TRPV3 ion channel without activating other TRPs (Xu et al., 2002; Moqrich et al., 2005). Flavors derived from oregano, clove, and thyme can activate the TRPV3 in the tongue (Xu et al., 2006). A second ion channel, TRPV4, has been found to respond to temperature thresholds of 24 and 34°C in human embryonic kidney (HEK) cells (Güler et al., 2002; Watanabe et al., 2002). TRPV1 is involved in the mediation of “heat” pain and is activated by temperatures above 43°C, as well as by irritants like capsaicin and chili peppers (Caterina et al., 1997; Jordt and Julius, 2002; Kwon et al., 2021).

Therefore, it is evident that the perception of temperature caused by chemical agents has a molecular foundation. Nevertheless, the thermal perception induced by certain chemicals can be utilized in the development of thermal displays following a comprehensive investigation into their impact on the human body. In a study by Hamazaki et al. (2022), capsaicin and menthol-induced thermal perception were used to elicit the thermal grill illusion among participants.

## 10 Thermal displays

Thermal displays are devices that can present desired thermal stimuli to the skin by different methods, including conduction, convection, and radiation of heat. Some of the displays covered in this section can provide heat feedback as well as other sensory cues like tactile information. Several psychophysical investigations into thermal displays were carried out with the goal of developing more efficient and effective thermal displays, which will be discussed in this section.

### 10.1 Psychophysics for thermal display design

The selection of design parameters for thermal or haptic displays should be grounded in psychophysical experiments. Jones and Singhal (2018) reviewed several psychophysical experiments to understand the interactions between the tactile and thermal sensory modalities in the context of a multisensory cutaneous display. The primary aim of the study was to ascertain the optimal method for delivering thermal cues using a thermal display, with the intention of identifying key attributes that may be included in a haptic device. According to Jones and Singhal (2018), the maximum temperature range for a thermal display should be 20°C, specifically ranging from 22 to 42°C. The ideal temperature resolution for heating and cooling in a thermal display is 0.001 and 0.002°C, correspondingly. The optimal range for the quantity of thermal elements inside an array is typically between 2 and 10. Finally, the recommended temporal transient resolutions for cooling and warming, as proposed by Jones and Singhal (2018), are 20 and 10°C/s, respectively.

Several psychophysical investigations have been conducted to examine the impact of temperature on electrotactile stimulation, a phenomenon that holds potential for enhancing the design of electrotactile thermal displays. Guo et al. (2019) observed that the voltage threshold of an electrovibration tactile display can be manipulated by temperature. Three voltage frequencies (25, 140, and 485 Hz) were applied to three surface temperatures (38°C, 30°C, and 18°C) to measure the absolute and discrimination thresholds of electrovibration stimuli. Results indicated that a cold stimulus increased the absolute threshold for high-frequency voltage. However, the temperature had no significant effect on the discrimination thresholds of the electrovibration stimuli (Guo et al., 2019). According to Ray and Manivannan (2022), the application of thermal stimulation has been observed to contribute to a decrease in the threshold for electrotactile perception by around 13%–17%. There is an evident requirement for conducting more rigorous psychophysical investigations aimed at establishing thermal display design guidelines.

### 10.2 Classification of thermal displays

There are different methods of thermally stimulating the skin. Conduction (thermo-electric heat pumps), convection (air and liquid), radiation (IR and microwave), or combinations of these methods can be implemented to provide thermal stimulation (Jones and Berris, 2002). Examples of thermal displays developed using different methods are discussed below (Table 3).

**Table 3:** Classification and examples of thermal displays.

**Conduction-based thermal displays**	**Convection + conduction-based thermal displays**	**Radiation-based thermal displays**
WeaRelaxAble by Klamet et al. (2016) •This wearable device, controlled using a wristband, applies thermal stimulation (using Peltier devices, TECI-1703) along with audio, light, and vibration to provide relaxation from stress. Thermal stimulation was done in the shoulder, loin, and groin areas. •The participants reported the cold stimulation as a “strange sensation,” whereas warm stimulation was reported as “pleasing.” Heat-Nav by Tewell et al. (2017) •This thermal array display (TAD) used three Peltier devices (MCPE1-01708NCS, Multicomp) to provide thermal feedback on the forearm for navigation through a 2D maze. The two temperatures used were 35 and 29°C, and the device was controlled by Arduino •Warm stimuli was used to signal “right direction,” whereas cold stimuli signaled “wrong direction” ThermalBracelet by Peiris et al. (2019) •This bracelet thermally stimulated (heated and cooled) the skin around the wrist at six locations using thermal modules made of Peltier devices (KSMH029F, KELK Ltd.) and temperature sensors •Spatio-temporal patterns of stimulation were used to provide haptic notifications and alerts Skin-like thermo-haptic (STH) device by Lee et al. (2020) •This skin-like, soft, and stretchable device elicited desirable thermal sensation (ranging from 15 to 45°C) with high controllability for VR-based applications •The thermo-haptic device was made of alternating p- and n-type bismuth telluride thermoelectric pellets interconnected by polyimide-coated Cu electrodes and encapsulated with thermally conductive elastomer Thermal display glove by Kim et al. (2020) •This silicon (Ecoflex 00-30) glove was embedded with piezoelectric sensors (made of polyvinylidene fluoride) for hand tracking and highly flexible and durable thermoelectric devices for thermal stimulus to improve realism in the VR environment •This thermoelectric device is made of n- and p-type thermoelectric legs solder pasted on Cu electrodes that are coated with polyimide substrate Thermoelectric tactile display (TTD) by Oron-Gilad et al. (2008) •This display elicited the thermal grill illusion on the forearm using Peltier devices (TEC, SH0.1-23-06L, Melcor) to serve as a tactile language •This display was comprised of six components: Thermal Actuator Unit (TAU), Galvanic Skin Response (GSR), Voltage Control Unit (VCU), PC Controller, NI 9211 (National Instruments), and Thermo-Couple (TC) sensors.	Pump-Actuated Thermal Compression Haptic (PATCH) Device by Goetz et al. (2020) •This soft wearable device used water of varying temperatures to present pressure and temperature cues (ranging from 17 to 42°C) for VR-based applications •Hot and cold tanks supplied water to four actuator pouches of the device using circulation pumps and valves, which was then returned to the tanks by a peristaltic pump PDMS-based flexible display by Gallo and Bleuler (2015) •This display performed hydraulic actuation using water of controlled temperature to provide multimodal (tactile and thermal) haptic information in teleoperation devices •The tactile module was made of a flexible polydimethylsiloxane (PDMS) body with four tactile cells, whereas the thermal module consisted of a Peltier element, a heatsink, a thermistor, and a heat spreader Water-based display by Hayakawa et al. (2015) •This display controlled the flow and temperature of water to use it as the medium for heat transfer to a presentation unit made of vinyl chloride resin that was attached to a chair. Water flow was controlled using pumps (KP-501T, Koshin LTD) •Water flow was controlled using pumps (KP-501T, Koshin LTD), and the temperature was controlled by mixing hot and cold water using solenoid valves (CKD Corp.). To increase realism, the temperature was synchronized with different images Water-based display by Honaga et al. (2015) •The temperature of this display was regulated by controlling the volume of water flow using a pump (KP-103, KOSHIN) •The water was circulated from a tank to thermal displays of varying sizes and then back to the tank using a pump Mesh-fabric gloves by Kamigaki et al. (2020) •These cotton gloves use airborne ultrasound phased arrays (AUPAs) to produce non-contact thermal stimulation in the hands without any Peltier elements to provide alerts or notifications in critical situations •Two modes of irradiation were applied, namely, static pressure (constant amplitude ultrasound) and amplitude modulation (ultrasound modulated at 150 Hz)	HeatHapt by Saga (2015) •This device focused halogen lamp light on the skin using a pan/tilt mirror to evoke the desired thermal sensation •Ultrasonic range sensors were used to measure the hand's position and a thermal camera was used to measure the temperature of the hand that was maintained at 45°C VR Thermal Kit by Dionisio (1997) •This device used Infrared lamps to thermally stimulate the skin of the user sitting on a chair to create the sensation of a virtual hell. •The kit also included ventilators and Peltier devices to thermally stimulate the skin using convection and radiation

The thermal displays developed in previous studies have been classified into three categories based on the method employed for thermal transmission. The first category includes thermal displays in which thermal transmission takes place through conduction. The second category belongs to thermal displays, which use both convection and conduction. The third category consists of thermal displays where radiation is used to heat up the skin.

#### 10.2.1 Conduction-based thermal displays

Conduction-based displays are in contact with the skin and can directly thermally stimulate the thermoreceptors. Peltier devices or thermoelectric coolers (TEC) have been used to design thermal devices for applications in virtual reality (Caldwell et al., 1995; Dionisio, 1997). The WeaRelaxAble by Klamet et al. (2016) implemented combinations of various kinds of feedback stimuli to enhance the stress resistance of the user. Thermal stimulation was presented to the groin, loin, and shoulder regions of the body. The participants reported the warming stimulus as relaxing and pleasant. Tewell et al. (2017) created the Heat-Nav, which uses three Peltier elements to provide direction (heating for the correct direction and cooling for the incorrect direction) for navigating a two-dimensional maze. Peiris et al. (2019) developed a smartwatch-integratable ThermalBracelet that can stimulate six locations around the wrist using Peltier elements. The ThermalBracelet was developed to provide haptic guidance and haptic notification using spatio-temporal stimulation (Peiris et al., 2019). The thermoelectric tactile display (TTD) by Oron-Gilad et al. (2008) uses thermoelectric coolers (TECs) to generate the thermal grill illusion (TGI) on the forearm. Apart from these traditionally used Peltier devices, modified versions of such devices are also implemented in thermal displays.

Thermoelectric devices were designed to be more flexible in order to achieve a wider application. A skin-like, soft, and stretchable thermo-haptic device developed by Lee et al. (2020) was used for wearable VR applications. The skin-like thermo-haptic (STH) device ran on a feedback control algorithm and could instantaneously heat or cool deformable skin surfaces to mimic a desirable thermal sensation. Unlike most thermal management devices, the STH can work in bi-functional mode (heating and cooling mode) (Lee et al., 2020). Kim et al. (2020) developed a thermal display glove for VR applications that was also highly flexible. The silicon glove was comprised of piezoelectric sensors and customized flexible thermoelectric devices (TEDs). The sensors tracked the hand posture from the finger joints, and the TEDs provided heating or cooling to three fingers (index finger, middle finger, and thumb) and the upper part of the palm (Kim et al., 2020). Similar use of illusions in thermo-tactile displays can help manipulate the tactile and temperature perception of the user.

#### 10.2.2 Convection + conduction-based thermal displays

Some thermal displays present thermal stimuli to the skin using a combination of convection and conduction. Water or air are mostly employed to carry the heat by convection to the display which is in contact with the skin. Goetz et al. (2020) created a Pump-Actuated Thermal Compression Haptic (PATCH) Device that can present thermal and compression cues simultaneously for potential application in a haptic-VR system such as entertainment, gaming, virtual communication, medical applications, and therapy. The PATCH actuators can be filled or emptied with water of varying temperatures to create pressure and temperature cues ranging from 17 to 42°C (Goetz et al., 2020). A flexible display developed by Gallo and Bleuler (2015) can reproduce the pulse-like thermal feedback in the master device of surgical robots. The device was based on polydimethylsiloxane (PDMS) and used hydraulic actuation for the pulse. The temperature of the water used for hydraulics was controlled to provide the desired thermal feedback (Gallo and Bleuler, 2015). Another display developed by Hayakawa et al. (2015) used water as a medium to transfer heat. The flow of water was controlled and the temperature change was synchronized with images to provide a high-speed temperature display on the neck. The water-based display by Honaga et al. (2015) controlled the volume of water flow using a pump to control the temperature of the display. Apart from using water, cold air has also been used to stimulate the skin. In a study by Nakajima et al. (2019), cold mist and halogen light were focused on the skin to elicit the thermal grill illusion. Ultrasound-phased arrays of 40 kHz directed the cold air toward the skin using acoustic streaming. This contributed to the formation of a cold spot with a temperature of around 27.7°C on the skin (Nakajima et al., 2019).

Apart from streaming air flow, ultrasound arrays can cause heating of the skin. In a recent study, Kamigaki et al. (2020) developed a method where mesh-fabric gloves were used as non-contact thermal and vibrotactile displays by focused airborne ultrasound irradiation. Airborne ultrasound phased arrays (AUPAs) were used to produce thermal stimulation at a focal point on the glove. The cotton gloves absorbed the ultrasound and produced vibrotactile and thermal stimulation when the irradiation patterns were changed (Kamigaki et al., 2020).

#### 10.2.3 Radiation-based thermal displays

Radiation is also used as a non-contact approach by several thermal displays to present temperature stimuli. Saga (2015) proposed a haptic display named HeatHapt, which uses thermal radiation to heat the skin. Light from a Halogen Lamp was focused onto the skin by controlling the tilt of a mirror, which caused a heat sensation or a nociceptive sensation. The VR Thermal Kit by Dionisio (1997) uses infrared lamps to create an immersive experience of a virtual hell.

### 10.3 Applications of thermal displays

Thermal displays possess a diverse range of applications, encompassing virtual reality (VR) and communication, among others. The increasing popularity of virtual reality (VR) is evident as it finds application in several domains for the purpose of simulating various scenarios. Numerous thermal displays have been previously devised with the objective of enhancing realism in virtual reality (VR) experiences (Goetz et al., 2020; Kim et al., 2020; Lee et al., 2020). In VR, thermal displays can be used to simulate the material qualities of virtual items like a cold can or a hot cup. Similar applications can also be made for telemarketing. Thermal displays, in addition to their use in the virtual world, can be a useful communication tool.

Displays have been designed to convey information to the user, whether for notification, alarm, or instruction (Peiris et al., 2019). In crucial situations such as rescue operations, thermo-tactile displays can be used to transmit a range of information by building a thermo-tactile language (Oron-Gilad et al., 2008; Wilson, 2013). Similarly, thermal displays can be utilized to give vital information directly to surgical gloves during surgery or to send danger alerts when near dangerous regions in workplaces (Kamigaki et al., 2020). Teleoperation is a rapidly developing field with a wide range of potential applications in areas such as nuclear reactors, surgical operation beds, and calamities. A teleoperating device with a thermal display can increase the accuracy of handling the device's components (Shin and Chang, 2021).

A collaboration of art and technology was explored in a study by Kushiyama et al. (2006) in which “Thermoesthesia” was discussed. “Thermoesthesia” is an artwork where the users interact with the art through thermal displays. Some possible approaches to bringing illusions to thermal displays have been described in the following sections.

## 11 Future directions in introducing thermal illusions into thermal displays

Although various efforts were made to create a thermal display, it is evident that further research is needed in this area. Thermal displays need to become more compact, portable, precise, and efficient. Thermal illusions could provide new approaches to building thermal displays that are more resource-efficient.

### 11.1 Displaying material properties using thermal illusions

In areas like virtual reality and telemarketing, thermal displays can be employed to depict the material qualities of virtual objects. Such simulations can be manipulated by illusions such as the temperature texture illusion or the temperature weight illusion. In telemarketing, providing a more realistic feel of the virtual goods might result in a more satisfactory buying experience for customers, resulting in fewer product returns.

### 11.2 Displaying pain using thermal illusions

The thermal grill illusion (TGI) can be used to represent pain as a burning or pinching sensation (Bouhassira et al., 2005). Using non-harmful stimuli and alternating warm and cool displays can elicit a painful experience. These displays can be employed not only in virtual reality environments but also in research related to chronic pain management.

### 11.3 Influencing localization of stimuli using thermal illusions

As discussed in the previous section, thermal referral can induce a thermal sensation (warm or cold) in a body area where no such stimulus exists (Green, 1977b). The ability to localize thermal stimuli can also be altered in the thermal spatio-temporal illusion by altering the time periods between the stimuli (Singhal and Jones, 2016b). Such thermal illusions can aid in the development of more efficient thermal displays that use fewer stimulators. By adjusting the time duration between stimuli, thermal stimulation can be perceived in body regions where no such stimuli are presented.

### 11.4 Enhancing thermal perception using thermal illusions

It is obvious from studying illusions that other sense modalities, such as vision, have a role in temperature perception as well. The mere sight of something warm or cold touching a fake body part might evoke a thermal perception in visual thermal illusion (VTI) (Kanaya et al., 2012) and Butcher's tongue illusion (Michel et al., 2014). Effectively combining visual and thermal cues can enhance the user's perception and result in improved thermal displays.

### 11.5 Communication using thermal illusions

Information can be transmitted in a multitude of ways using multisensory haptic displays. Illusions can help build a language based on tactile and thermal perception that can be displayed. An array of thermal displays embedded in a wearable device can be used to present spatial-temporal illusions. This interaction between time duration and thermo-tactile stimuli can aid in the formation of a haptic language for notification display. Furthermore, illusions like the thermal grill illusion can cause minor pain to convey emergency alerts directly to the skin, allowing the user to avoid hazardous terrain.

### 11.6 Displaying temperature using chemical agonists

As seen in Chemical thermal illusions, the effects of certain chemical compounds can also cause thermal perceptions. Chemical substances can be used to elicit or enhance a certain thermal sense in thermal displays. However, before using such compounds in thermal displays, it is necessary to investigate their effects on the skin as well as the display.

## 12 Discussion

A better understanding of thermal illusions can not only deepen our knowledge of thermal perception but also help us manipulate thermal perception to simulate desired sensations using thermal displays.

### 12.1 Gap analysis

Numerous research studies have been conducted to elucidate the underlying mechanisms driving thermal illusions and their effects on thermal perception. However, there are still ambiguities regarding the mechanisms of some of these thermal illusions. For instance, there are multiple theories about thermal grill illusion (TGI), and investigations are necessary to establish a comprehensive model of TGI. Similarly, for thermal referral, the mechanism involving homogenization and then localization of thermal sensation needs to be supported by mathematical models. Modeling of several other thermal illusions is also necessary to predict their perceptual outcomes. Such modeling will be possible through rigorous neurophysiological and psychophysical investigations.

Illusions also provide a doorway to multimodal interactions. Temperature weight illusion, temperature texture illusion, and wetness illusion are examples of illusions where the temperatures affect the material properties (weight, texture, and wetness) of objects. Although it is known that temperature stimulates certain mechanoreceptors, further studies are needed to investigate the extent of thermo-tactile interaction. Similarly, visual thermal illusion shows how vision can sometimes dominate touch. However, vision might not always dominate touch, and further studies are needed to understand the visuo-tactile interactions as well.

Thermal displays are still far from being perfect and there is a huge scope for improvement in thermal displays and multimodal displays in general. The existing thermal displays mostly implement Peltier devices to transfer heat to the skin through conduction. Since thermal sensation has poor spatial acuity, the number of thermal stimulators that should be used for a certain application should be well studied. Moreover, not much attempt has been made to incorporate perceptual illusions to present desired sensations and optimize the thermal displays. Psychophysical studies with thermal displays are needed to set the design parameters and also to observe the effect of thermal illusions on the performance of those displays.

Thermal displays should take into account properties such as thermal adaptation, spatial summation, or the rate of temperature change affecting the thermal detection. The mode of heat transfer (conduction, convection, or radiation) might also influence the perceptual qualities of thermosensation and hence should be considered while setting the design specification for thermal displays.

### 12.2 Summary

Extensive studies have been conducted on the perception of temperature. Research from multiple perspectives, such as psychophysics and electrophysiology, has intended to present a clearer picture. Thermal illusions are a major contributor to the understanding of thermosensation and its interaction with other sensory modalities. By manipulating certain aspects of thermosensation, one's perception of weight, texture, space, or time can be controlled. In fact, certain arrangements of non-painful temperature stimuli have the ability to produce an illusory effect of pain. Studying these thermal illusions might help in discovering other illusions related to temperature perception, which can in turn enhance our knowledge of thermal perception.

It is clear that the current understanding of the mechanisms behind these thermal illusions is not robust. There are ambiguities between several theories of these illusions. Attempts have been made to formulate mathematical models for some perceptual interactions. However, more studies are definitely required to present a clearer perspective. For instance, it is evident from illusions such as the temperature weight illusion and the temperature texture illusion that temperature plays an important role in the activity of mechanoreceptors. Apart from the interactions between tactile and temperature perception, there are illusions that show that visual information also impacts the perception of temperature. Also, illusions such as the spatio-temporal illusion show that temporal information or the perception of time can influence the perception of the location of thermal stimuli. Hence, illusions point to the fact that the thermal sensory modality is interdependent on other sensory modalities, and it is necessary that these sensations be studied together to understand their interactions.

It can be concluded that thermal illusions are an active area of research and a lot of studies will be required to dissect the underlying mechanisms of most of the illusions. The more they are studied, the more clarity is brought regarding the sensory perception of temperature in our body. However, these findings can immensely help in designing better thermal displays and thermal devices for virtual reality applications. Thermal displays can be used not only for material simulation but also for conveying abstract concepts and the use of thermal perception in interactive art (Jones and Ho, 2008). Thermal displays have their applications not only in gaming but also in tele-existing robots, telemarketing, etc. It can open new doors for communication in the future and hence should be studied with the utmost severity and rigor. Moreover, thermal displays can be used in medical research and treatments. Incorporating the effects of thermal illusions can greatly enhance the experience of the users and also the designs of thermal displays. Very few thermal displays have utilized this aspect of using thermal illusions for thermal displays. As a result, there is a lot of room for fresh research, discoveries, and innovations in this exciting field of thermal illusions and displays.

## Author contributions

SK: Conceptualization, Software, Visualization, Writing – original draft, Writing – review & editing. AK: Supervision, Validation, Visualization, Writing – review & editing. MM: Resources, Supervision, Validation, Writing – review & editing.
